# Energy Metabolism and Lipidome Are Highly Regulated during Osteogenic Differentiation of Dental Follicle Cells

**DOI:** 10.1155/2022/3674931

**Published:** 2022-07-16

**Authors:** Oliver Pieles, Marcus Höring, Sadiyeh Adel, Torsten E. Reichert, Gerhard Liebisch, Christian Morsczeck

**Affiliations:** ^1^Department of Oral and Maxillofacial Surgery, University Hospital Regensburg, Franz-Josef-Strauss-Allee 11, 93053 Regensburg, Germany; ^2^Institute of Clinical Chemistry and Laboratory Medicine, University Hospital Regensburg, Franz-Josef-Strauss-Allee 11, 93053 Regensburg, Germany

## Abstract

Dental follicle cells (DFCs) are stem/progenitor cells of the periodontium and give rise to alveolar osteoblasts. However, understanding of the molecular mechanisms of osteogenic differentiation, which is required for cell-based therapies, is delimited. This study is aimed at analyzing the energy metabolism during the osteogenic differentiation of DFCs. Human DFCs were cultured, and osteogenic differentiation was induced by either dexamethasone or bone morphogenetic protein 2 (BMP2). Previous microarray data were reanalyzed to examine pathways that are regulated after osteogenic induction. Expression and activity of metabolic markers were evaluated by western blot analysis and specific assays, relative amount of mitochondrial DNA was measured by real-time quantitative polymerase chain reaction, the oxidative state of cells was determined by a glutathione assay, and the lipidome of cells was analyzed via mass spectrometry (MS). Moreover, osteogenic markers were analyzed after the inhibition of fatty acid synthesis by 5-(tetradecyloxy)-2-furoic acid or C75. Pathway enrichment analysis of microarray data revealed that carbon metabolism was amongst the top regulated pathways after osteogenic induction in DFCs. Further analysis showed that enzymes involved in glycolysis, citric acid cycle, mitochondrial activity, and lipid metabolism are differentially expressed during differentiation, with most markers upregulated and more markedly after induction with dexamethasone compared to BMP2. Moreover, the cellular state was more oxidized, and mitochondrial DNA was distinctly upregulated during the second half of differentiation. Besides, MS of the lipidome revealed higher lipid concentrations after osteogenic induction, with a preference for species with lower numbers of C-atoms and double bonds, which indicates a de novo synthesis of lipids. Concordantly, inhibition of fatty acid synthesis impeded the osteogenic differentiation of DFCs. This study demonstrates that energy metabolism is highly regulated during osteogenic differentiation of DFCs including changes in the lipidome suggesting enhanced de novo synthesis of lipids, which are required for the differentiation process.

## 1. Introduction

Periodontal diseases are a common health issue that can lead to severe tissue damage and tooth loss. Tissue engineering based on biomaterials and stem cells is a promising approach for regeneration of functional periodontal tissue [1–3]. Dental follicle cells (DFCs) are stem/progenitor cells of the periodontium and give rise to the alveolar bone, cementum, and periodontal ligament during tooth development. Thus, DFCs are suitable for stem cell-based therapies of periodontal diseases [4, 5]. Furthermore, they can be easily isolated from extracted wisdom teeth [6]. However, besides much progress in the recent years, the molecular mechanisms of differentiation processes into functional tissue cells are still insufficiently understood, which impedes their optimal use for targeted therapies [5, 7].

The osteogenic differentiation of DFCs can be induced in vitro by stimulating the cells with dexamethasone or bone morphogenetic protein 2 (BMP2) amongst others [8, 9]. Several studies indicate that the two inducers target different molecular signaling pathways. For example, BMP2 drives the differentiation via the expression of runt-related transcription factor 2 (RUNX2) and distal-less homeobox 3 (DLX3), while dexamethasone-induced osteogenesis requires other transcription factors like zinc finger and BTB domain-containing 16 (ZBTB16) [10–12]. However, the two differentiation procedures also share similarities like the fact that classical isoforms of protein kinase C (PKC) disturb osteogenesis with both inducers [13]. Besides particular molecular pathways, only little attention has been spent to the role of general cellular metabolism for DFC differentiation. A previous study showed that osteogenesis of DFCs is sensitive to changes in AMP-activated protein kinase (AMPK) activity [14], which is a key molecular sensor for cellular energy [15]. It is presumable that energy metabolism might play a crucial role for osteogenic differentiation of precursor cells. Bone marrow-derived mesenchymal stem cells (MSCs) were reported to activate oxidative phosphorylation, but not glycolysis, after osteogenic induction [16, 17], and hypoxia inhibits the differentiation [18]. Reactive oxygen species (ROS), which are mainly generated by mitochondrial metabolism, can regulate the osteogenic differentiation [19]. Mitochondrial activity was further shown to stimulate the osteogenic differentiation via acetylation of *β*-catenin [20]. Beyond, also metabolism of cellular lipids participates in energy homeostasis and is implicated in several molecular pathways like activation of PKC [21–23].

In order to further unravel the molecular mechanisms behind the osteogenic differentiation of DFCs, this study is aimed at analyzing the energy metabolism including markers for glycolysis, citric acid cycle, oxidative phosphorylation, and lipid metabolism after induction of differentiation with dexamethasone and BMP2. Moreover, the lipidome of differentiating DFCs and the role of fatty acid synthesis were analyzed in this study.

## 2. Materials and Methods

### 2.1. Cell Culture

Human dental follicle cells were purchased from AllCells (Alameda, CA, USA) and cultured in Dulbecco's Modified Eagle's Medium (DMEM) containing 10% fetal bovine serum (FBS) and 1% penicillin (100 U/ml)/streptomycin (100 *μ*g/ml) solution (all Sigma-Aldrich, St. Louis, MO, USA) with 37°C and 5% CO_2_ in humidified atmosphere. Growth medium was changed three times in a week. Cells until passage 8 were used for the experiments.

### 2.2. Osteogenic Differentiation

For induction of osteogenic differentiation, subconfluent DFCs were treated with osteogenic differentiation medium consisting of DMEM supplemented with 2% FBS, antibiotics as above, 20 mM HEPES, 10 mM *β*-glycerophosphate, 100 *μ*M phospho-ascorbic acid, and 100 nM dexamethasone (all Sigma-Aldrich), or with BMP2 differentiation medium which contains 50 ng/ml BMP2 (Biomol, Hamburg, Germany) in place of dexamethasone. As control, cells were simultaneously cultured in DMEM with 2% FBS and antibiotics as above. The differentiation media were changed twice in a week.

### 2.3. Microarray Analysis of Transcriptome

Publicly available microarray data (GSE20963) were used for analysis. The data include the gene expression profile of DFCs that were treated with different osteogenic inducers including dexamethasone and BMP2 for seven days compared to control medium in biological triplicates for each group. Data were processed as described earlier [24]. Processed gene expression data were used to search for regulated genes after osteogenic induction: The genes selected were up- or downregulated with at least factor 2 and with a *p* value below 0.05 in Student's *t*-test by both dexamethasone and BMP2. A list of the selected genes is available in Supplementary Table 1. Afterwards, a pathway enrichment analysis was performed with the selected genes with DAVID bioinformatics resources [25, 26] using the KEGG (Kyoto Encyclopedia of Genes and Genomes) pathway database [27–29]. A *p* value was determined with the EASE score, and pathways with a *p* value below 0.05 were considered significantly addressed.

### 2.4. Western Blot Analysis

DFCs were lysed and scraped in a buffer consisting of 20 mM trisHCl (pH 8.0), 137 mM NaCl, 48 mM NaF, 1% (*v*/*v*) NP-40, 10% (*v*/*v*) glycerol, 2 mM Na_3_VO_4_, phosphatase inhibitor cocktail 3 (Sigma-Aldrich), and cOmplete™ mini protease inhibitor cocktail (1 tablet in 10 ml, Sigma-Aldrich) in H_2_O. Supernatants were collected after centrifugation of the lysates at 14,000 rpm for 5 min at 4°C, and the protein concentration was determined with the Pierce™ BCA Protein Assay Kit (Thermo Fisher Scientific, Waltham, MA, USA). Subsequently, lysates were mixed with the Laemmli sample buffer (BIO-RAD, Hercules, CA, USA) before boiling for 5 min at 95°C. Samples were loaded onto a 4–15% Mini-PROTEAN® TXG StainFree™ Protein Gel (BIO-RAD), and proteins were separated by sodium dodecyl sulfate polyacrylamide gel electrophoresis (SDS-PAGE). After activation by UV light, proteins were transferred onto Amersham Protran® 0.2 *μ*m nitrocellulose membranes (Sigma-Aldrich) using either a Trans-Blot® SD Semi-Dry Transfer Cell or a Trans-Blot® Turbo™ Transfer System (both BIO-RAD). Photographs of total lane protein were taken with a ChemiDoc™ Touch Imaging System (BIO-RAD). Membranes were then washed in Tris-buffered saline (TBS) for 5 min before blocking in 5% bovine serum albumin (BSA) in Tween-containing TBS (TBST) for 1 hour at room temperature. Subsequently, the membranes were incubated overnight at 4°C in a primary antibody against hypoxia-inducible factor 1-alpha (HIF-1*α*), cytochrome c, prohibitin 1, cytochrome c oxidase subunit 4 (COX IV), pyruvate dehydrogenase, succinate dehydrogenase complex subunit A (SDHA), heat shock protein 60 (HSP60), voltage-dependent anion channel (VDAC), hexokinase I, hexokinase II, phosphofructokinase, glycerinaldehyd-3-phosphat-dehydrogenase (GAPDH), pyruvate kinase M1/2, pyruvate kinase M2, lactate dehydrogenase A, aconitase 2, isocitrate dehydrogenase 1, isocitrate dehydrogenase 2, dihydrolipoamide succinyltransferase (DLST), fumarase, citrase synthase, mitochondrial pyruvate carrier 1, mitochondrial pyruvate carrier 2, acetyl-CoA carboxylase, phospho-acetyl-CoA carboxylase (Ser79), ATP-citrate lyase, phospho-ATP-citrate lyase (Ser455), acetyl-CoA synthetase, acyl-CoA synthetase, fatty acid synthase (all Cell Signaling Technology, Danvers, MA, USA), or elongation of very long chain fatty acids protein 6 (ELOVL6) (Abcam, Cambridge, UK). The primary antibodies were all diluted 1 : 1000 in TBST with 5% BSA. Afterwards, membranes were washed three times in TBST for 10 min each, before incubation in the secondary antibody horseradish peroxidase- (HRP-) linked anti-rabbit IgG (Cell Signaling Technology), which was diluted 1 : 1000 in TBST with 5% skimmed milk powder for 1 hour at room temperature. Thereupon, membranes were washed twice in TBST for 10 min each, once in phosphate-buffered saline (PBS) for at least 5 min and once in TBS for 5 min, before a chemiluminescent signal was developed by using the Clarity™ western ECL substrate (BIO-RAD) and recorded with a ChemiDoc™ Touch Imaging System (BIO-RAD). Densitometric quantification was performed with the software Image Lab version 6.0.1 (BIO-RAD) with normalization of protein bands to total lane protein and the control group.

### 2.5. L-Lactate Assay

The Glycolysis Cell-Based Assay Kit (Cayman Chemical, Ann Arbor, MI, USA) was used according to the manufacturer's handbook. Briefly, DFCs in a 96-well cell culture plate with 200 *μ*l medium per well were centrifuged, and 20 *μ*l of supernatant was used to determine the amount of l-lactate in a colorimetric reaction, which was measured spectrophotometrically at OD = 490 nm.

### 2.6. Hexokinase Activity Assay

The Hexokinase Colorimetric Assay Kit (Sigma-Aldrich) was used according to manufacturer's instructions. Briefly, DFCs were washed with PBS, scraped in assay buffer from the kit, and centrifuged. The supernatant was further used to determine hexokinase activity in a colorimetric reaction, which was measured spectrophotometrically at OD = 450 nm in kinetic mode.

### 2.7. Malate Dehydrogenase Activity Assay

The Malate Dehydrogenase Activity Assay Kit (Abcam) was used according to the manufacturer's protocol booklet. Briefly, cells were washed with PBS and scraped in assay buffer from the kit. After centrifugation, supernatants were used to determine malate dehydrogenase activity in a colorimetric reaction, which was measured spectrophotometrically at OD = 450 nm in kinetic mode.

### 2.8. Complex I Activity Assay

Mitochondria were isolated from DFCs with the Mitochondria Isolation Kit for Cultured Cells (Abcam) according to manufacturer's instructions. Afterwards, the Complex I Enzyme Activity Microplate Assay Kit (Abcam) was used to determine complex I activity following the manufacturer's protocol booklet. Briefly, proteins from isolated mitochondria were extracted using the detergent solution from the kit and loaded onto a microplate precoated with complex I antibodies. After 3 hours incubation, the microplate wells were washed, before the assay solution from the kit was added in order to initiate a colorimetric reaction, which was measured spectrophotometrically at OD = 450 nm in kinetic mode.

### 2.9. Real-Time Quantitative Polymerase Chain Reaction (qPCR)

The QIAamp® DNA Mini Kit (Qiagen, Hilden, Germany) was used to isolate DNA from DFCs. For this, the “Protocol for Cultured Cells” in the manufacturer's handbook was used. Both trypsinization and scraping were applied to detach the adherent cells from the culture plates. The qPCR analysis was performed with the SsoAdvanced™ Universal SYBR Green Supermix (BIO-RAD) and primers against mitochondrial DNA (forward primer: 5′-GCCTTCCCCCGTAAATGATA-3′, reverse primer: 5′-TTATGCGATTACCGGGCTCT-3′) or B2M (forward primer: 5′-TGCTGTCTCCATGTTTGATGTATCT-3′, reverse primer: 5′-TCTCTGCTCCCCACCTCTAAGT-3′) as reference for genomic DNA. The qPCR was run on a StepOnePlus™ Real-Time PCR System (Thermo Fisher Scientific) with the following protocol: polymerase activation for 2 min at 95°C, then 40 cycles each consisting of a denaturation step for 15 sec at 95°C, and an annealing/elongation step for 30 sec at 62°C, then melt curve analysis (for amplicon control) starting at 95°C for 15 sec, then 1 min at 60°C following temperature increase in 0.3°C steps for each 15 sec until 95°C. The amount of mitochondrial DNA was normalized to the amount of B2M genomic DNA and to the control group at day 1 by using the *ΔΔ*Ct method [30]. The qPCR protocol including primer sequences was adopted from Venegas and Halberg [31].

### 2.10. Glutathione Assay

The GSH/GSSG-Glo™ Assay kit (Promega, Madison, WI, USA) was used according to manufacturer's instructions to determine relative amounts of oxidized and reduced glutathione in DFCs. Briefly, cells were washed with PBS and lysed in a Glutathione Lysis Reagent from the kit for analysis of either oxidized or total glutathione. The amount of glutathione was then quantified using a luciferase reporter system. For each experimental group, three biological replicates were used for determination of oxidized and total glutathione, respectively. The amount of oxidized from total glutathione, which is used as indicator of oxidative stress, was normalized to the control group.

### 2.11. Mass Spectrometry Analysis of Lipidome (MS)

DFCs were washed with PBS and scraped in 0.2% sodium dodecyl sulfate (SDS) and cOmplete™ mini protease inhibitor cocktail (1 tablet in 5 ml, Sigma-Aldrich) in H_2_O. The protein concentration in the lysates was measured with the Pierce™ BCA Protein Assay Kit (Thermo Fisher Scientific). The samples were stored at -80°C until analysis. Cell homogenates were subjected to lipid extraction according to the method of Bligh and Dyer [32] in the presence of not naturally occurring lipid species as internal standards. The following lipid species were added as internal standards: PC 14 : 0/14 : 0, PC 22 : 0/22 : 0, PE 14 : 0/14 : 0, PE 20 : 0/20 : 0 (diphytanoyl), PS 14 : 0/14 : 0, PS 20 : 0/20 : 0 (diphytanoyl), PI 17 : 0/17 : 0, LPC 13 : 0, LPC 19 : 0, LPE 13 : 0, Cer d18:1/14 : 0, Cer d18 : 1/17 : 0, D7-FC, CE 17 : 0, CE 22 : 0, TG 51 : 0, TG 57 : 0, DG 28 : 0, and DG 40 : 0. The chloroform phase was recovered by a pipetting robot (Tecan Genesis RSP 150) and vacuum dried. The residues were dissolved in either 10 mM ammonium acetate in methanol/chloroform (3 : 1, *v*/*v*) (for low mass resolution tandem mass spectrometry) or chloroform/methanol/2-propanol (1 : 2 : 4, *v*/*v*/*v*) with 7.5 mM ammonium formate (for high-resolution mass spectrometry).

Mass spectrometry analysis of lipids was performed by direct flow injection analysis (FIA) using a triple quadrupole mass spectrometer (FIA-MS/MS; QQQ triple quadrupole) and a hybrid quadrupole-Orbitrap mass spectrometer (FIA-FTMS; high mass resolution).

FIA-MS/MS (QQQ) was performed in positive ion mode using the analytical setup and strategy described previously [33]. A fragment ion of *m*/*z* 184 was used for lysophosphatidylcholine (LPC) [34]. The following neutral losses were applied: phosphatidylethanolamine (PE) 141, phosphatidylserine (PS) 185, phosphatidylglycerol (PG) 189, and phosphatidylinositol (PI) 277 [35]. PE-based plasmalogens (PE P) were analyzed according to the principles described by Zemski Berry and Murphy [36]. Sphingosine-based ceramides (Cer) and hexosylceramides (HexCer) were analyzed using a fragment ion of *m*/*z* 264 [37]. Quantification was achieved by calibration lines generated by addition of naturally occurring lipid species to the respective sample matrix. Calibration lines were generated for the following naturally occurring species: PC 34 : 1, 36 : 2, 38 : 4, 40 : 0, and PC O-16 : 0/20 : 4; SM 18 : 1; O2/16 : 0, 18 : 1, and 18 : 0; LPC 16 : 0, 18 : 1, and 18 : 0; PE 34 : 1, 36 : 2, 38 : 4, and 40 : 6; PE P-16 : 0/20 : 4; PS 34 : 1, 36 : 2, 38 : 4, and 40 : 6; Cer 18 : 1; O2/16 : 0, 18 : 0, 20 : 0, 24 : 1, 24 : 0; and FC, CE 16 : 0, 18 : 2, 18 : 1, and 18 : 0.

The Fourier transform mass spectrometry (FIA-FTMS) setup is described in detail in Höring et al. [38]. Triglycerides (TG), diglycerides (DG), and cholesteryl ester (CE) were recorded in positive ion mode FTMS in range *m*/*z* 500-1000 for 1 min with a maximum injection time (IT) of 200 ms, an automated gain control (AGC) of 1 × 10^6^, three microscans, and a target resolution of 140,000 (at *m*/*z* 200). Phosphatidylcholine (PC) and sphingomyelin (SM) were measured in a negative ion mode with a mass range *m*/*z* 520-960. Multiplexed acquisition (MSX) was used for the [M+NH_4_]^+^ of free cholesterol (FC) (*m*/*z* 404.39) and D7-cholesterol (*m*/*z* 411.43) 0.5 min acquisition time, with a normalized collision energy of 10%, an IT of 100 ms, AGC of 1 × 10^5^, isolation window of 1 Da, and a target resolution of 140,000 [39]. Data processing details were described in Höring et al. [38] using the ALEX software [40] which includes peak assignment and intensity picking. The extracted data were exported to Microsoft Excel 2016 and further processed by self-programmed Macros. FIA-FTMS quantification was performed by multiplication of the spiked IS amount with the analyte-to-IS ratio.

The lipid species were annotated according to the latest proposal for shorthand notation of lipid structures that are derived from mass spectrometry [41]. For QQQ glycerophospholipid species, annotation was based on the assumption of even numbered carbon chains only. Concentration data are available in the supplementary file.

### 2.12. Inhibition of Fatty Acid Synthesis

In order to analyze the role of fatty acid synthesis on osteogenic differentiation, DFCs were treated with 5 *μ*g/ml fatty acid synthesis inhibitors C75 or 5-(tetradecyloxy)-2-furoic acid (TOFA, both Sigma-Aldrich) in differentiation or control medium. The corresponding medium with 0.05% (*v*/*v*) DMSO was used as vehicle control.

### 2.13. Reverse Transcription—Quantitative Polymerase Chain Reaction (RT-qPCR)

The RNeasy® Plus Mini Kit (Qiagen) was used to isolate RNA from DFCs. Concentration and purity of RNA were analyzed using a NanoDrop™ 2000 spectrophotometer (Thermo Fisher Scientific). Next, the iScript™ cDNA Synthesis Kit (BIO-RAD) was used to convert mRNA into cDNA (reverse transcription). The qPCR analysis was performed with the SsoAdvanced™ Universal Probes Supermix (BIO-RAD) and probe-containing PrimePCR™ primers (BIO-RAD) against GAPDH, RUNX2, and COL1A2 (Collagen type 1 alpha 2 chain) on a StepOnePlus™ Real-Time PCR System (Thermo Fisher Scientific) with the following protocol: 2 min polymerase activation at 95°C, then 40 cycles of 5 sec denaturation at 95°C, and 30 sec annealing/elongation at 60°C. Target gene expression was normalized to the expression of GAPDH and to the DMEM control group by use of the *ΔΔ*Ct method [30].

### 2.14. Alkaline Phosphatase (ALP) Activity Assay

For examining the ALP activity in DFCs, the cells were washed with PBS and lysed with Triton-X (0.1% in PBS). A portion of the lysate was pipetted in a fresh 96-well plate in order to measure the protein concentration using the Pierce™ BCA Protein Assay Kit (Thermo Fisher Scientific). Besides, p-nitrophenylphosphate (Sigma-Aldrich) was added to the remaining lysates, which then incubated at 37°C for 60 min. Immediately thereafter, the conversion into yellow p-nitrophenol was quantified spectrophotometrically at OD = 415 nm. The ALP activity values were normalized to the total protein of each sample and to the DMEM control group.

### 2.15. Alizarin Red Staining

Mineralization of extracellular matrix was analyzed by Alizarin Red staining. Cells were washed with PBS and fixed with 4% formalin. Subsequently, cells were washed with H_2_O three times, stained with Alizarin Red Solution (Merck Millipore, Billerica, MA, USA) for 20 min, and washed again with H_2_O three times. Microscopic photographs were then taken and staining was quantified by dissolving Alizarin Red crystals in cetylpyridinium chloride (10% in PBS) and measuring spectrophotometrically at OD = 595 nm. The obtained values were normalized to the DMEM control group.

### 2.16. Statistical Analysis

Experiments were performed in biological triplicates unless otherwise stated. Student's *t*-test and one-way analysis of variance (ANOVA) were performed with the software SPSS version 25 (IBM, Armonk, NY, USA) as described in the figure legends. *p* values < 0.05 were considered statistically significant and labelled as indicated in the figure legends.

## 3. Results

### 3.1. Carbon Metabolism Plays a Major Role during Osteogenic Differentiation of DFCs

Aiming at further unraveling the molecular mechanisms behind osteogenesis of DFCs, former microarray data were reanalyzed. The KEGG pathway analysis was performed to determine pathways that are significantly regulated after both osteogenic induction with dexamethasone and induction with BMP2. Amongst pathways related to biosynthesis and metabolism of proteins, carbon metabolism was significantly addressed (Table 1), which confirms that energy metabolism plays a major role during osteogenesis.

### 3.2. Glycolysis and Citric Acid Cycle Are Regulated during Osteogenic Differentiation of DFCs

**Figure 1:**
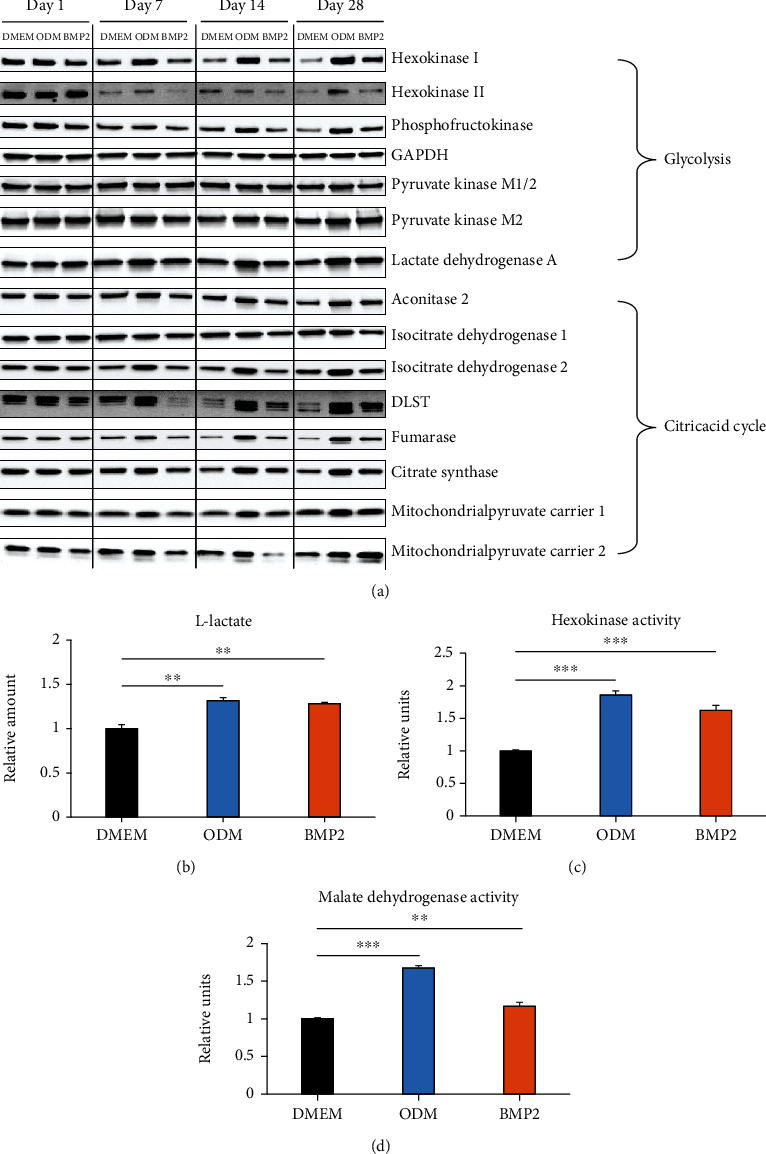
Expression of glycolysis and citric acid cycle markers during osteogenic differentiation of DFCs. (a) DFCs were cultured in osteogenic differentiation medium (ODM), BMP2 differentiation medium, or control medium (DMEM) for one, seven, 14, and 28 days before protein expression of the glycolysis markers hexokinase I, hexokinase II, phosphofructokinase, glycerinaldehyd-3-phosphat-dehydrogenase (GAPDH), pyruvate kinase M1/2, pyruvate kinase M2, and lactate dehydrogenase A, and protein expression of the citric acid cycle markers aconitase 2, isocitrate dehydrogenase 1, isocitrate dehydrogenase 2, dihydrolipoamide succinyltransferase (DLST), fumarase, citrase synthase, mitochondrial pyruvate carrier 1, and mitochondrial pyruvate carrier 2 were determined by western blot analysis. Quantification results and statistical analysis are shown in the Supplementary Table 2. (b–d) DFCs were cultured in osteogenic differentiation medium (ODM), BMP2 differentiation medium, or control medium (DMEM) for seven days before the amount of produced L-lactate (b) and hexokinase activity (c) were determined as markers of glycolysis and malate dehydrogenase activity was measured (d) as marker of citric acid cycle. Results are shown as means + standard deviation, and Student's *t*-test was performed to compare differentiation medium with control medium. ^∗∗^*p* < 0.01, ^∗∗∗^*p* < 0.001.

Hence, we investigated the signaling pathways involved in carbon metabolism, which is highly correlated with the energy metabolism of cells, more precisely. Starting with the glycolysis, we analyzed the expression of the most important enzymes by western blot analysis (Figure 1(a), upper part, and Supplementary Table 2). Protein expression of hexokinase I, hexokinase II, phosphofructokinase, and lactate dehydrogenase A was upregulated after osteogenic induction and more distinctly with dexamethasone, except for lactate dehydrogenase A, which was more upregulated by BMP2. In contrast, GAPDH and pyruvate kinase were expressed rather consistently. Additionally, the amount of produced L-lactate (Figure 1(b)) and hexokinase activity (Figure 1(c)) were significantly enhanced during osteogenic differentiation, which was again more pronounced with dexamethasone. Moreover, enzymes of the subsequent citric acid cycle were examined (Figure 1(a) lower part and Supplementary Table 2). The western blot analysis showed that expression of aconitase 2, isocitrate dehydrogenase 2, DLST, fumarase, and citrate synthase was upregulated at later stages of osteogenesis. In contrast, the enzymes isocitrate dehydrogenase 1, mitochondrial pyruvate carrier 1, and mitochondrial pyruvate carrier 2 were expressed rather consistently. Besides the protein expression of citric acid cycle markers, the activity of malate dehydrogenase was significantly upregulated during osteogenic differentiation, with a higher increase after induction with dexamethasone (Figure 1(d)).

### 3.3. Mitochondrial Metabolism Is Highly Regulated during Differentiation

**Figure 2:**
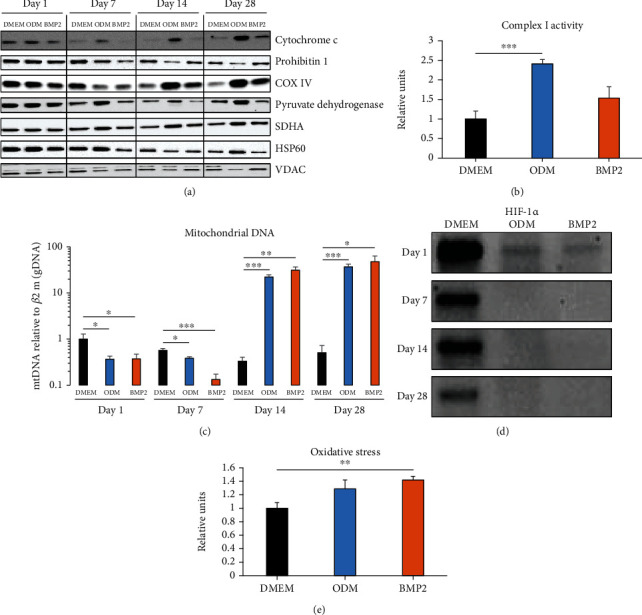
Evaluation of mitochondrial metabolism during osteogenic differentiation of DFCs. (a) DFCs were cultured in osteogenic differentiation medium (ODM), BMP2 differentiation medium, or control medium (DMEM) for one, seven, 14, and 28 days before protein expression of cytochrome c, prohibitin 1, cytochrome c oxidase subunit 4 (COX IV), pyruvate dehydrogenase, succinate dehydrogenase complex subunit A (SDHA), heat shock protein 60 (HSP60), and voltage-dependent anion channel (VDAC) was determined by western blot analysis. Quantification results and statistical analysis are shown in the Supplementary Table 2. (b) DFCs were cultured in osteogenic differentiation medium (ODM), BMP2 differentiation medium, or control medium (DMEM) for seven days before complex I activity was determined in isolated mitochondria. (c, d) DFCs were cultured in osteogenic differentiation medium (ODM), BMP2 differentiation medium, or control medium (DMEM) for one, seven, 14, and 28 days before the amount of mitochondrial DNA relative to genomic DNA was measured by qPCR analysis (c) and protein expression of HIF-1*α* was determined by western blot analysis (*n* = 1, d). (e) DFCs were cultured in osteogenic differentiation medium (ODM), BMP2 differentiation medium, or control medium (DMEM) for seven days. The amount of oxidized glutathione from total glutathione was measured as indicator for oxidative stress. Results are shown as means + standard deviation, and Student's *t*-test was performed to compare differentiation medium with control medium at the same time point (b, c, e). ^∗^*p* < 0.05, ^∗∗^*p* < 0.01, ^∗∗∗^*p* < 0.001.

Subsequently, we investigated the regulation of mitochondrial markers during osteogenic differentiation of DFCs (Figure 2). We found that protein expression of cytochrome c, pyruvate dehydrogenase, and SDHA was distinctly upregulated, while the expression of HSP60 was slightly and not significantly upregulated, and the expression of prohibitin 1 and VDAC was downregulated after induction with dexamethasone. In contrast, these markers were less regulated or even downregulated after BMP2 induction until day 14, while the expression of cytochrome c, prohibitin 1, and SDHA was significantly upregulated at day 28. Besides, protein expression of COX IV was downregulated after seven days and upregulated at the later time points with both osteogenic inducers (Figure 2(a) and Supplementary Table 2). Additionally, complex I activity in DFCs was enhanced after seven days osteogenic differentiation, but only significantly with dexamethasone treatment (Figure 2(b)). The relative amount of mitochondrial DNA in DFCs was regulated similarly to COX IV expression: At days 1 and 7 during differentiation, relative amount of mitochondrial DNA was significantly reduced, while significantly upregulated at days 14 and 28 with both inducers (Figure 2(c)). Besides, protein expression of HIF-1*α* was inhibited at all investigated time points (Figure 2(d)), which suggests that more oxygen species are present in the cells. Concordantly, a higher proportion of oxidized glutathione was found in DFCs seven days after osteogenic induction (Figure 2(e)), which indicates higher oxidative stress.

### 3.4. Lipid Metabolism Is Regulated during Osteogenic Differentiation of DFCs

**Figure 3:**
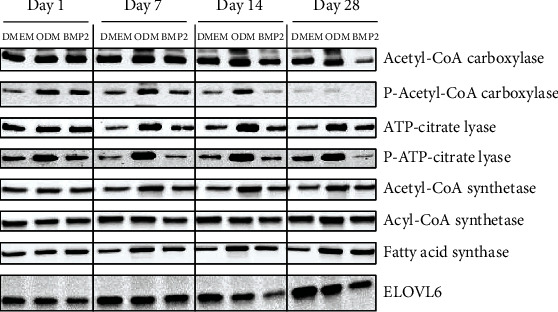
Expression of lipid metabolism markers during osteogenic differentiation of DFCs. DFCs were cultured in osteogenic differentiation medium (ODM), BMP2 differentiation medium, or control medium (DMEM) for one, seven, 14, and 28 days before protein expression of acetyl-CoA carboxylase, phospho-acetyl-CoA carboxylase, ATP-citrate lyase, phospho-ATP-citrate lyase, acetyl-CoA synthetase, acyl-CoA synthetase, fatty acid synthase, and elongation of very long chain fatty acids protein 6 (ELOVL6) was determined by western blot analysis. Quantification results and statistical analysis are shown in the Supplementary Table 2.

**Figure 4:**
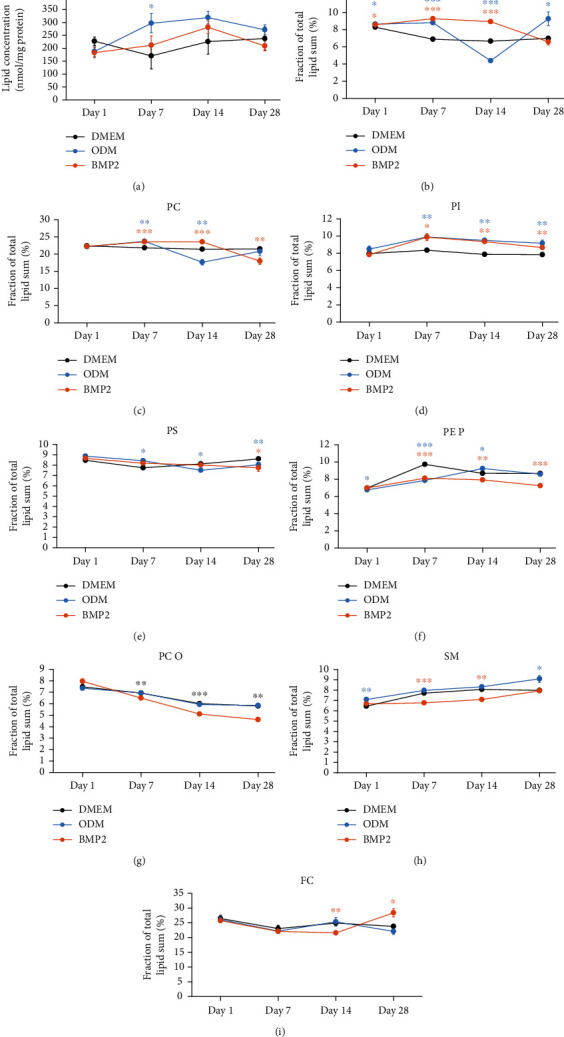
Regulation of membrane lipids during osteogenic differentiation of DFCs. DFCs were cultured in osteogenic differentiation medium (ODM), BMP2 differentiation medium, or control medium (DMEM) for one, seven, 14 and 28 days before the lipidome was analyzed by MS. This figure shows the regulation of total lipids (a) and the fraction of phosphatidylethanolamines (PE, b), phosphatidylcholines (PC, c), phosphatidylinositols (PI, d), phosphatidylserines (PS, e), phosphatidylethanolamine-based plasmalogens (PE P, f), phosphatidylcholine-ethers (PC O, g), sphingomyelins (SM, h), and free cholesterol (FC, i). Data points show means ± standard deviation, and Student's *t*-test was performed to compare differentiation medium with control medium at the same time point. ^∗^*p* < 0.05, ^∗∗^*p* < 0.01, ^∗∗∗^*p* < 0.001. Asterisks relate to the differentiation medium with data points and line in same color.

Next, we explored the metabolism of lipids as another major type of carbon-based molecules in cells. Initially, the expression of relevant enzymes involved in lipid metabolism was analyzed by western blot analysis (Figure 3 and Supplementary Table 2). The results show weak—and not significant—upregulation of ATP-citrate lyase and fatty acid synthase and significant upregulation of acetyl-CoA-synthetase by both osteogenic inducers. However, expression of phosphorylated ATP-citrate lyase was only upregulated by dexamethasone and tendentially downregulated by BMP2, which indicates a higher enzyme activation state in dexamethasone-treated DFCs [42]. Besides, the expression of acetyl-CoA carboxylase is reduced at days 14 and 28, while no significant upregulation of the Ser79 phosphorylation was observed at the later time points. Instead, Ser79-phosphorylated acetyl-CoA carboxylase is upregulated by dexamethasone until day 14, however not significantly, which is an indicator for inhibition of this enzyme [43]. At day 28, almost no phosphorylated acetyl-CoA carboxylase was detected in any sample. Additionally, expression of ELOVL6 markedly increased in all culture media until day 28, most distinctly in DMEM control medium, while expression of acyl-CoA synthetase was unaltered during differentiation. We next analyzed the lipidome of differentiating DFCs by MS. In the overview, the sum of all lipids was slightly enhanced—more pronounced with dexamethasone—during osteogenesis from day 7 (Figure 4(a)). However, some particular lipid classes were more regulated than others. Analyzing the proportions of the most abundant classes of membrane lipids revealed that especially the proportion of phosphatidylethanolamines (PE) varied during differentiation (Figure 4(b)). Moreover, phosphatidylcholines (PC) showed a similar regulation pattern, but less distinctly (Figure 4(c)). Interestingly, the fraction of PE and PC was markedly decreased in ODM-treated cells at day 14 compared to days 7 and 28. In addition, the proportion of phosphatidylinositols (PI) was steadily upregulated during osteogenic differentiation from day 7 (Figure 4(d)). Besides, the lipid classes phosphatidylserines (PS), PE-based plasmalogens (PE P), PC-ethers (PC O), sphingomyelins (SM), and free cholesterol (FC) were less regulated (Figures 4(e)–4(i)). Regulation patterns of less abundant lipid classes are shown in Supplementary Figure S1.

### 3.5. The Composition of Lipid Species Is Altered during Osteogenic Differentiation

**Figure 5:**
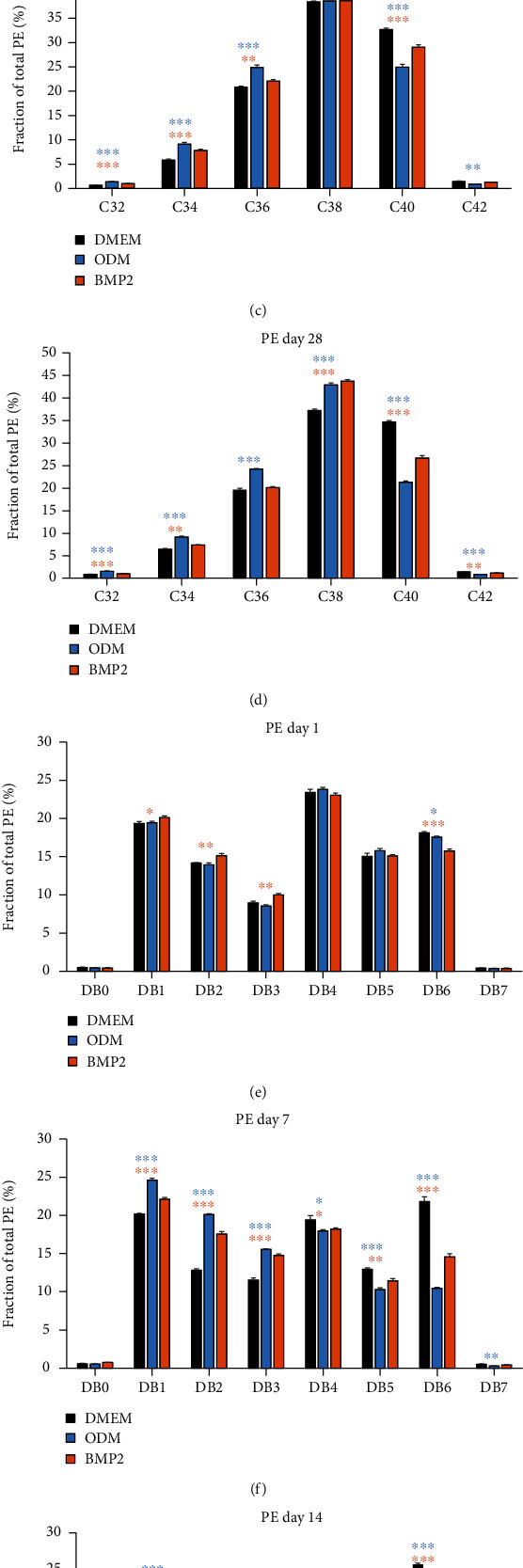
Regulation of phosphatidylethanolamines with different numbers of C-atoms and double bonds during osteogenic differentiation of DFCs. DFCs were cultured in osteogenic differentiation medium (ODM), BMP2 differentiation medium, or control medium (DMEM) for one, seven, 14, and 28 days before the lipidome was analyzed by MS. This figure shows the regulation of phosphatidylethanolamines (PE) with different C-atom numbers at day 1 (a), day 7 (b), day 14 (c), and day 28 (d), and PEs with different numbers of double bonds (DB) at day 1 (e), day 7 (f), day 14 (g), and day 28 (h). Results are shown as means + standard deviation, and Student's *t*-test was performed to compare differentiation medium with the control medium at the same time point. ^∗^*p* < 0.05, ^∗∗^*p* < 0.01, ^∗∗∗^*p* < 0.001. Asterisks relate to the differentiation medium with bars in same color.

In order to analyze the regulation of lipids in more detail, we investigated the composition of PEs—as highly regulated membrane lipid—during osteogenic differentiation and found a heterogeneous regulation of PEs with different numbers of C-atoms and double bonds (Figure 5). While almost no changes were detected at day 1 (Figures 5(a) and 5(e)), lipid species were highly regulated at day 7: PEs with 32, 34, and 36 C-atoms and with one, two, or three double bonds were distinctly upregulated, which was accompanied by a decrease of PEs with 40 C-atoms and polyunsaturated PEs with four, five, or six double bonds (Figures 5(b) and 5(f)). The same regulation pattern was observable at day 14 (Figures 5(c) and 5(g)). More precisely, PE species 32 : 1, 34 : 1, 34 : 2, 36 : 2, 36 : 3, 38 : 2, and 38 : 3 were upregulated at days 7 and 14 and PE 40 : 3 only at day 14, while the species 38 : 6, 40 : 5, and 40 : 6 were downregulated at both days 7 and 14, PE 36 : 1, 38 : 1, and 40 : 4 only at day 7, and PE 38 : 4 and 38 : 5 only at day 14 (Supplementary Figure S2). It is worth noting that the changes were usually more pronounced in dexamethasone-induced DFCs compared to osteogenic induction with BMP2. At day 28, the composition of PE species in dexamethasone-induced cells was similar to days 7 and 14, with the only difference that PEs with 38 C-atoms were upregulated additionally. In BMP2-treated DFCs, PEs with 38 C-atoms were also elevated, as well as PEs with two, three, or four double bonds, while species with 40 C-atoms and with five or six double bonds were downregulated at day 28 (Figures 5(d) and 5(h)).

### 3.6. Storage Lipids and Diacylglycerol Are Highly Regulated during the Osteogenic Differentiation of DFCs

**Figure 6:**
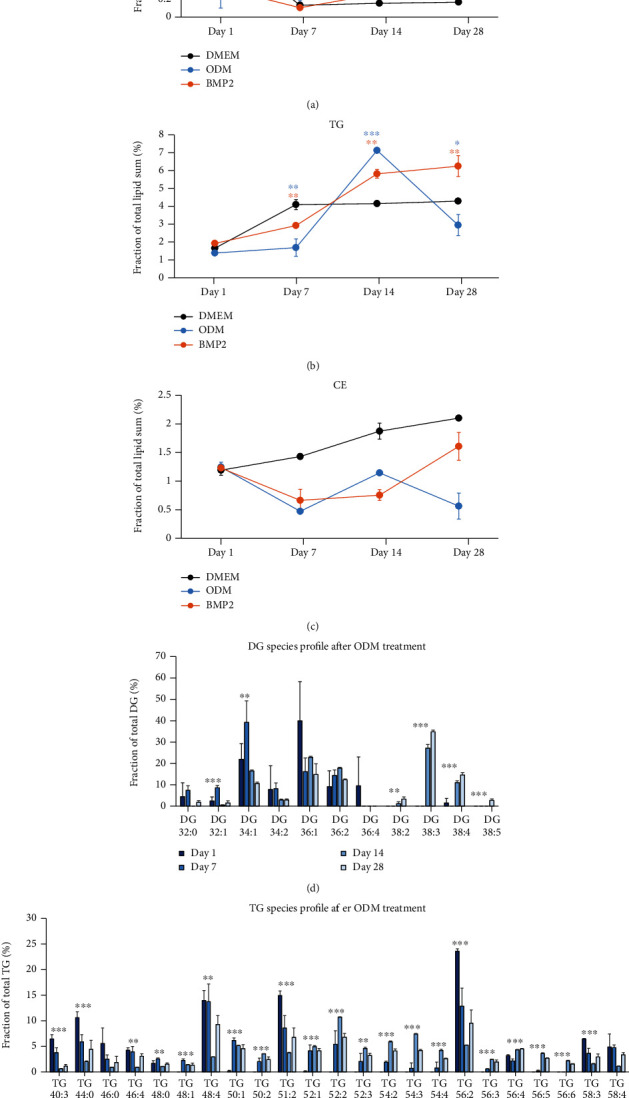
Regulation of storage lipids and diacylglycerol during osteogenic differentiation of DFCs. DFCs were cultured in osteogenic differentiation medium (ODM), BMP2 differentiation medium, or control medium (DMEM) for one, seven, 14, and 28 days before the lipidome was analyzed by MS. (a–c) The line charts show the fraction of diacylglycerols (DG, a), triacylglycerols (TG, b), and cholesteryl esters (CE, c). Data points show means ± standard deviation, and Student's *t*-test was performed to compare differentiation medium with control medium at the same time point. ^∗^*p* < 0.05, ^∗∗^*p* < 0.01, ^∗∗∗^*p* < 0.001. Asterisks relate to the differentiation medium with data points and line in same color. (d, e) Furthermore, the composition of DG species (d) and TG species (e) in cells treated with ODM at the different time points is shown. Only species with an abundance of more than 2% for at least one time point are shown as means + standard deviation, and one-way ANOVA was performed to determine significant differences in the fraction of a specific species at different time points. ^∗∗^*p* < 0.01, ^∗∗∗^*p* < 0.001.

Furthermore, we investigated the regulation of storage lipids and diacylglycerols (DG), as DGs are precursors for triacylglycerols (TG) and glycerophospholipids, during the osteogenic differentiation of DFCs (Figure 6). The proportion of DGs was initially decreased compared to the control medium at day 1, while it was steadily increasing at the later time points and more distinctly in dexamethasone-treated cells (Figure 6(a)). Regarding the regulation of triacylglycerols, a decrease in compare to control medium is observable later, namely, at day 7, while upregulated at days 14 and 28, except for dexamethasone-treated DFCs, where the proportion of TGs is decreased again at day 28 (Figure 6(b)). By contrast, cholesteryl esters (CE) are constantly downregulated compared to control medium from day 7 after osteogenic induction (Figure 6(c)). As the DG fraction is highly increased in dexamethasone-treated DFCs, we investigated the DG species profile at the different time points in detail (Figure 6(d)). The results show that monounsaturated DGs with 34 and 36 C-atoms are the most abundant species at days 1 and 7, while polyunsaturated DGs with 38 C-atoms are dominant at days 14 and 28. We also observed the species profile of TGs in dexamethasone-treated cells and concordantly found more species with lower numbers of C-atoms and double bonds at the earlier time points, while polyunsaturated species with a higher number of C-atoms were more abundant at days 14 and 28 (Figure 6(e)).

### 3.7. Inhibition of Fatty Acid Synthesis Impedes the Osteogenic Differentiation of DFCs

**Figure 7:**
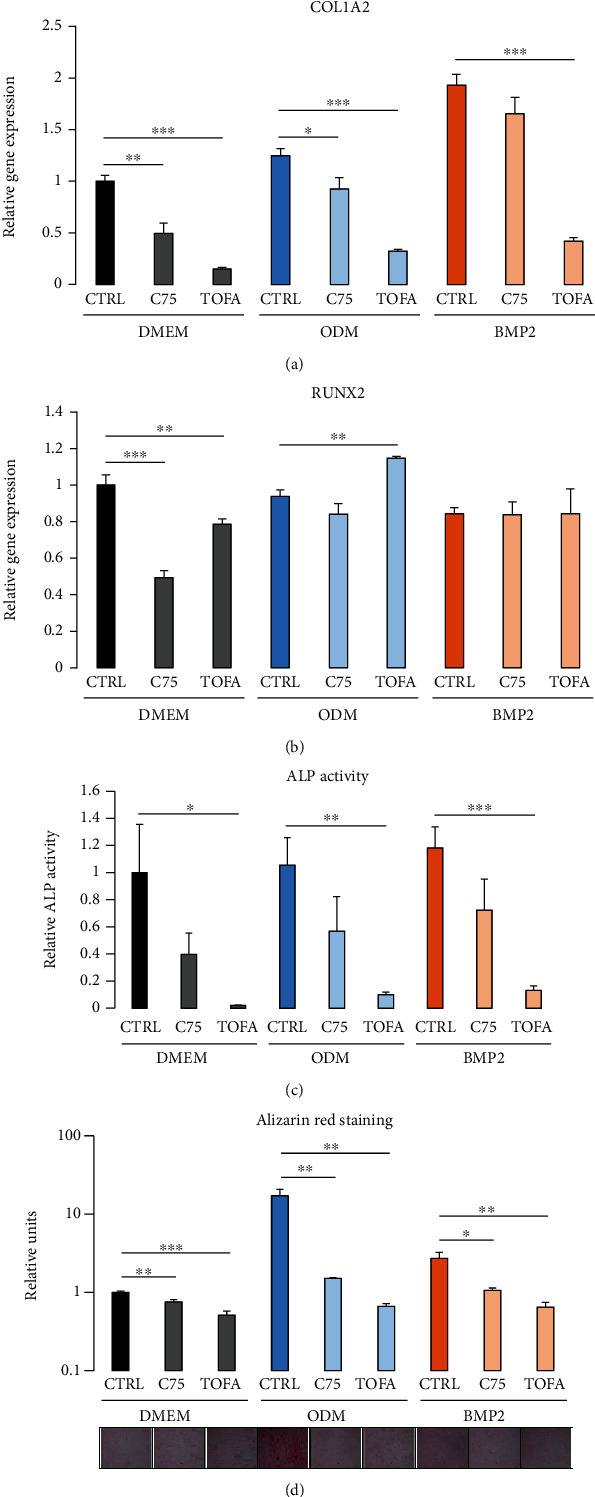
Regulation of osteogenic differentiation in DFCs after inhibition of fatty acid synthesis. DFCs were cultured in osteogenic differentiation medium (ODM), BMP2 differentiation medium, or control medium (DMEM) and simultaneously treated with 5 *μ*g/ml fatty acid synthesis inhibitors TOFA or C75 or vehicle control for different time periods before evaluation of osteogenic differentiation markers: (a, b) Gene expressions of COL1A2 (a) and RUNX2 (b) relative to gene expression of GAPDH were determined after three days treatment by RT-qPCR. (c) Activity of alkaline phosphatase (ALP) relative to total protein was measured after seven days treatment. (d) Mineralization of extracellular matrix was determined by Alizarin Red staining after 28 days treatment. Photographic pictures of the staining are shown below the bar chart which shows the relative quantification results as means + standard deviation. Student's *t*-tests were performed to compare inhibitor treatment with the vehicle control. ^∗^*p* < 0.05, ^∗∗^*p* < 0.01, ^∗∗∗^*p* < 0.001.

As lipid concentrations were elevated after osteogenic induction, further experiments were performed investigating the role of fatty acid synthesis for the osteogenic differentiation of DFCs. Cells were treated with either TOFA or C75, which act as inhibitors of fatty acid synthesis by inhibiting the enzymes acetyl-CoA carboxylase or fatty acid synthase, respectively [44–46]. Treating DFCs with TOFA resulted in a higher phosphorylation of acetyl-CoA carboxylase, which indicates inactivation of the enzyme [43], while C75 treatment reduced the expression of fatty acid synthase (Supplementary Figure S3). Subsequent analysis of osteogenic markers showed that gene expression of COL1A2 as well as ALP activity and Alizarin Red staining were reduced after inhibitor treatment, more distinctly and throughout significantly with TOFA (Figures 7(a), 7(c), and 7(d)). However, gene expression of RUNX2 was only reduced by fatty acid synthesis inhibition in DMEM control medium, while unaltered or even elevated in the differentiation media (Figure 7(b)).

## 4. Discussion

The results of this study showed that energy metabolism is highly regulated after osteogenic induction in DFCs. Against previous findings reporting that only oxidative phosphorylation and not glycolysis are upregulated [16], our experiments showed induction of some glycolysis markers as well. Presumably, glycolytic energy production is not dispensable for osteogenesis. Concordantly, a previous study showed that miR-34a suppresses the osteogenic differentiation of MSCs by the downregulation of lactate dehydrogenase A [47], which was upregulated during osteogenesis in DFCs. Nonetheless, mitochondrial markers were also regulated in DFCs and analysis of mitochondrial DNA copy number revealed a high upregulation in the second half of the differentiation process. This is accompanied by inhibition of HIF-1*α* expression and a more oxidized cellular state. Downregulation of HIF-1*α* was also reported during osteogenic differentiation of bone marrow-derived MSCs, while reactivation of HIF-1*α* inhibits oxidative phosphorylation [16]. Interestingly, inhibition of HIF-1*α* expression occurs directly after osteogenic induction in DFCs, while mitochondrial DNA is even downregulated at days 1 and 7. Probably, other molecular mechanisms are important for the induction of differentiation, while a sufficient energy supply and oxidative phosphorylation activity are necessary in later stages when premature osteoblasts lead to biomineralization of extracellular matrix. However, a higher energy demand is probably not the only purpose of enhanced mitochondrial activity during osteogenesis, since a previous study reported that inhibition of oxidative phosphorylation can indeed be compensated by glycolysis, which maintains the ATP level, but still impedes osteogenic differentiation [20]. Against this finding, induction of pseudohypoxia enhanced the proliferation and also the osteogenic differentiation of PDL cells while concurrently shifting energy metabolism from oxidative phosphorylation to glycolysis [48]. Hence, glycolytic metabolism is not necessarily related to impeded differentiation. Upregulation of mitochondrial activity is further reported to depend on Akt [49], which is highly regulated during osteogenesis in DFCs [13]. The results indicate that mitochondrial upregulation plays a pivotal and diverse role in the molecular clockwork of osteogenic differentiation and that energy metabolism affects osteogenesis in more respects than only ATP supply.

The results further revealed differences between dexamethasone and BMP2 induction. With regard to mitochondrial markers, it is noticeable that admittedly both inducers lead to upregulation of mitochondrial DNA copy number from day 14 but upregulate different proteins as dexamethasone predominantly enhanced expression of cytochrome c and pyruvate dehydrogenase, while BMP2 induced the expression of prohibitin 1. Differences in carbon metabolism comparing osteogenic induction with dexamethasone or BMP2 were also detected in MSCs [50]. The observations strengthen the hypothesis that both inducers lead to the same outcome—upregulation of mitochondrial activity, a more oxidative state, inhibition of HIF-1*α*, and finally matrix mineralization—but function via regulating different proteins and signaling pathways, which are worth to be further explored in future studies.

Analysis of enzymes concerning lipid metabolism also showed high regulation after osteogenic induction with both inducers including upregulation of some important markers for fatty acid metabolism. This is in line with the MS analysis of the lipidome which concurrently showed higher lipid concentrations. Overall, regulation of lipidome was similar with both osteogenic inducers, although more pronounced in dexamethasone-treated DFCs as the expression of related enzymes and the concentration of lipids were higher here. This is very interesting with regard to the previous observation that dexamethasone-induced mineralization is more intense compared to BMP2-treated DFCs [13]. The transcription factor ZBTB16, whose expression is highly induced by dexamethasone [11], might play a role for this difference as it is also related to energy metabolism [51]. A study on rabbit MSCs could further show that the lipidome changes during osteogenic differentiation of precursor cells and, moreover, that lipidomes are similar in cells from different origins [52]. This implies a fundamental role of lipid metabolism for osteogenesis. Detailed analysis with the example of PE species revealed that especially species with a number of C-atoms until 36—which mainly corresponds to PEs with two fatty acids with each 18 C-atoms—and a higher degree of saturation was increased at day 7, which indicates that de novo synthesis is the main source for elevated lipid levels. Concordantly, PE species with C-atom numbers until 38 and more double bonds were found until day 28, presuming chain elongation and desaturation following synthesis. This assumption is in accordance with the observation that expression of ELOVL6, which is known to elongate fatty acids with up to 16 C-atoms into species with up to 18 C-atoms [53], was increased over time.

Eventually, inhibition of fatty acid synthesis resulted in downregulation of most investigated osteogenic markers. Especially COL1A2 gene expression, ALP activity, and Alizarin Red staining were impacted, which are important markers for extracellular matrix production and mineralization. Besides, gene expression of RUNX2 was less affected. Interestingly, ALP activity and RUNX2 expression were barely upregulated in differentiation media compared with control medium. This implies that the DFCs used in our study already exhibit a high basal expression of these markers, which is probably associated with a high osteogenic potential. Thus, further regulation of RUNX2 and ALP might not be required to drive the differentiation process. However, as the inhibition of fatty acid synthesis impedes mineralization but not RUNX2 expression, the relevance of lipid metabolism for osteogenesis is likely not connected to RUNX2. Instead, de novo synthesis of fatty acids and lipids is presumably required for proper function of osteogenically induced DFCs to produce hard tissue. Fatty acid synthesis in eukaryotic cells occurs in both the cytoplasm and mitochondria [54]. Hence, enhanced mitochondrial activity might be an important driver for lipogenesis during the differentiation.

The definite role of enhanced lipid production for biomineralization remains elusive and probably has different functions for the mineralization process. Predominantly, cells might use fatty acids as energy source, which was also reported by a previous study [55]. This is further supported by the fact that the fraction of DGs was elevated from day 7, as intermediate products, and eventually TGs at day 14, whose role is predominantly defined as energy storage [56]. Concordantly, DG species 32 : 0, 32 : 1, and 34 : 1—presumably due to de novo synthesis—showed peaks at day 7 during dexamethasone-induced differentiation. However, DG species 38 : 3 and 38 : 4 and TG species 56 : 5 and 56 : 6 were highly induced at days 14 and 28. Presumably, upregulation of polyunsaturated species occurred due to uptake from the culture medium in order to enhance the cellular energy resources.

Interestingly, concentration of DGs at day 1 was lower in the differentiation media compared to control medium. As DGs are an important cofactor for activation of classical PKC isoforms [57], which inhibit the osteogenic differentiation of DFCs [13], downregulation of DGs right after osteogenic induction might be important to impede PKC activation. Furthermore, it is worth noting that cholesteryl esters are the only lipid class that is strictly downregulated during differentiation compared to control medium. By contrast, the proportion of free cholesterol is constantly high. Remaining a stable level of free cholesterol might play another pivotal role during differentiation. Previous studies showed that oxysterols—oxidized derivatives of cholesterol—support the osteogenic differentiation of stem cells and counteract negative impacts of oxidative stress [58, 59]. Thus, the abundance of free cholesterol together with enhanced mitochondrial activity might be an important mechanism to support the osteogenic differentiation of DFCs. Mitochondrial activity, a more oxidative cellular state and upregulation of free cholesterol together could be another mechanism that contributes to osteogenesis. Further research will be required to evaluate the significance of those pathways for osteogenesis.

## 5. Conclusion

Energy metabolism is highly regulated during osteogenic differentiation of DFCs. Several enzymes involved in glycolysis, citric acid cycle, mitochondrial respiratory chain, and fatty acid metabolism are upregulated upon osteogenic induction, more notably by dexamethasone, accompanied by a more oxidative cellular state. Eventually, analysis of lipidome showed higher lipid concentrations with essential changes in the composition of lipid species, while inhibition of fatty acid synthesis impeded osteogenesis. The results demonstrate that energy metabolism plays a vital role during the osteogenic differentiation of DFCs.

## Figures and Tables

**Table 1:** Pathway enrichment analysis of genes significantly regulated by dexamethasone and BMP2.

KEGG pathway	% of regulated genes	*p* value
Biosynthesis of amino acids	6.8	3.43*E* − 07
Glycine, serine, and threonine metabolism	4.5	3.12*E* − 05
Biosynthesis of antibiotics	7.5	1.70*E* − 04
Aminoacyl-tRNa biosynthesis	3.8	3.57*E* − 03
Carbon metabolism	4.5	4.41*E* − 03
Protein digestion and absorption	3.8	9.86*E* − 03
Arginine biosynthesis	2.3	1.54*E* − 02
Alanine, aspartate, and glutamate metabolism	2.3	4.40*E* − 02

## Data Availability

The lipidome concentration data are available as supplementary file. The other raw data are available from the authors on reasonable request.

## Abbreviations

AGC: Automated gain control
ALP: Alkaline phosphatase
AMPK: AMP-activated protein kinase
ANOVA: Analysis of variance
BMP2: Bone morphogenetic protein 2
BSA: Bovine serum albumin
CE: Cholesteryl ester
Cer: Ceramides
COL1A2: Collagen type 1 alpha 2 chain
COX IV: Cytochrome c oxidase subunit 4
DB: Double bonds
DFC: Dental follicle cell
DG: Diglycerides
DLST: Dihydrolipoamide succinyltransferase
DLX3: Distal-less homeobox 3
DMEM: Dulbecco's modified Eagle's medium
ELOVL6: Elongation of very long chain fatty acids protein 6
FBS: Fetal bovine serum
FC: Free cholesterol
FIA: Flow injection analysis
FTMS: Fourier transform mass spectrometry
GAPDH: Glycerinaldehyd-3-phosphat-dehydrogenase
HexCer: Hexosylceramides
HIF-1*α*: Hypoxia-inducible factor 1-alpha
HRP: Horseradish peroxidase
HSP60: Heat shock protein 60
IT: Injection time
KEGG: Kyoto Encyclopedia of Genes and Genomes
LPC: Lysophosphatidylcholine
LPE: Lysophosphatidylethanolamine
MS: Mass spectrometry
MSC: Mesenchymal stem cell
MSX: Multiplexed acquisition
ODM: Osteogenic differentiation medium
PBS: Phosphate-buffered saline
PC: Phosphatidylcholine
PC O: Phosphatidylcholine-ether
PE: Phosphatidylethanolamine
PE P: PE-based plasmalogens
PG: Phosphatidylglycerol
PI: Phosphatidylinositol
PKC: Protein kinase C
PS: Phosphatidylserine
qPCR: Real-time quantitative polymerase chain reaction
ROS: Reactive oxygen species
RUNX2: Runt-related transcription factor 2
SDHA: Succinate dehydrogenase complex subunit A
SDS: Sodium dodecyl sulfate
SDS-PAGE: Sodium dodecyl sulfate polyacrylamide gel electrophoresis
SM: Sphingomyelin
TBS: Tris-buffered saline
TBST: Tris-buffered saline with Tween20
TG: Triglycerides
TOFA: 5-(Tetradecyloxy)-2-furoic acid
VDAC: Voltage-dependent anion channel
ZBTB16: Zinc finger and BTB domain-containing 16

## Supplementary Materials

Supplementary 1 List of genes significantly regulated by dexamethasone and BMP2. Genes involved in the pathway carbon metabolism are in bold type.

Supplementary 2 Quantification results of western blots in Figures 1(a), 2(a), and 3 with means ± standard deviation and molecular weight of antigens. Statistically significant (*p* < 0.05) values compared to control medium (DMEM) at the same time point are in bold type.

Supplementary 3 Regulation of less abundant lipid classes during osteogenic differentiation of DFCs. DFCs were cultured in osteogenic differentiation medium (ODM), BMP2 differentiation medium, or control medium (DMEM) for one, seven, 14, and 28 days before the lipidome was analyzed by MS. This figure shows the fraction of lysophosphatidylethanolamines (LPE, a), lysophosphatidylcholines (LPC, b), ceramides (Cer, c), hexosylceramides (HexCer, d), and phosphatidylglycerols (PG, e). Data points show means ± standard deviation, and Student's *t*-test was performed to compare differentiation medium with control medium at the same time point. ^∗^*p* < 0.05, ^∗∗^*p* < 0.01, ^∗∗∗^*p* < 0.001. Asterisks relate to the differentiation medium with data points and line in same color.

Supplementary 4 Species profiles of phosphatidylethanolamines during osteogenic differentiation of DFCs. This figure shows the composition of phosphatidylethanolamine (PE) species analyzed by MS in DFCs cultured in osteogenic differentiation medium (ODM), BMP2 differentiation medium, or control medium (DMEM) at days 7 (a) and 14 (b). Results are shown as means + standard deviation, and Student's *t*-test was performed to compare differentiation medium with control medium at the same time point. ^∗^*p* < 0.05, ^∗∗^*p* < 0.01, ^∗∗∗^*p* < 0.001. Asterisks relate to the differentiation medium with bars in same color.

Supplementary 5 Inhibition of fatty acid synthesis by TOFA and C75 in DFCs. DFCs were treated with 5 *μ*g/ml fatty acid synthesis inhibitors TOFA (a) or C75 (b) or vehicle control for two hours (a) or one day (b) before protein expression of phospho-acetyl-CoA carboxylase (a) or fatty acid synthase (b) was determined by western blot analysis. Results are shown as means + standard deviation, and Student's *t*-test was performed to compare inhibitor treatment with the vehicle control. ^∗^*p* < 0.05.
