# Supplementary material for: Energy Metabolism and Lipidome Are Highly Regulated during Osteogenic Differentiation of Dental Follicle Cells
Source: Stem Cells Int. 2022 Jul 16;2022:3674931. doi: 10.1155/2022/3674931 (PMC9315453; doi:10.1155/2022/3674931)
Supplement: Supplementary 2 — Quantification results of western blots in Figures 1(a), 2(a), and 3 with means ± standard deviation and molecular weight of antigens. Statistically significant (p < 0.05) values compared to control medium (DMEM) at the same time point are in bold type. [file 3674931.f2.pdf]

Supplementary Table 2

Antigen	Molecular weight	DMEM 1d	ODM 1d	BMP2 1d	DMEM 7d	ODM 7d	BMP2 7d	DMEM 14d	ODM 14d	BMP2 14d	DMEM 28d	ODM 28d	BMP2 28d
Hexokinase I	102 kDa	1,000 ± 0,294	1,151 ± 0,249	0,901 ± 0,199	0,964 ± 0,104	1,402 ± 0,223	0,831 ± 0,185	0,584 ± 0,201	**1,203 ± 0,123**	0,543 ± 0,142	0,383 ± 0,115	**1,698 ± 0,285**	**1,469 ± 0,422**
Hexokinase II	102 kDa	1,000 ± 0,185	0,731 ± 0,106	0,869 ± 0,205	0,185 ± 0,064	0,251 ± 0,051	0,089 ± 0,039	0,240 ± 0,086	0,226 ± 0,094	0,213 ± 0,046	0,151 ± 0,054	0,300 ± 0,072	0,372 ± 0,248
Phosphofructo-kinase	80 kDa	1,000 ± 0,143	1,090 ± 0,091	0,943 ± 0,058	0,460 ± 0,072	0,611 ± 0,067	0,401 ± 0,036	0,320 ± 0,051	**0,512 ± 0,042**	0,304 ± 0,051	0,201 ± 0,034	**0,546 ± 0,084**	0,342 ± 0,075
GAPDH	37 kDa	1,000 ± 0,201	0,929 ± 0,057	1,050 ± 0,086	1,084 ± 0,058	0,892 ± 0,131	1,215 ± 0,294	0,999 ± 0,251	0,688 ± 0,123	1,074 ± 0,184	0,993 ± 0,254	0,787 ± 0,110	1,292 ± 0,299
Pyruvate Kinase M1/2	60 kDa	1,000 ± 0,268	1,030 ± 0,220	1,179 ± 0,274	1,436 ± 0,368	1,133 ± 0,174	1,693 ± 0,374	1,312 ± 0,300	0,882 ± 0,073	1,332 ± 0,202	1,198 ± 0,245	0,938 ± 0,087	1,168 ± 0,103
Pyruvate Kinase M2	60 kDa	1,000 ± 0,128	1,001 ± 0,084	0,981 ± 0,220	1,070 ± 0,179	0,993 ± 0,152	1,084 ± 0,274	0,912 ± 0,217	0,809 ± 0,023	0,950 ± 0,318	0,763 ± 0,303	0,986 ± 0,160	1,527 ± 0,294
Lactate Dehydrogenase A	37 kDa	1,000 ± 0,122	1,111 ± 0,138	1,119 ± 0,144	1,154 ± 0,089	1,235 ± 0,026	1,383 ± 0,124	1,119 ± 0,055	1,183 ± 0,084	**1,413 ± 0,102**	1,060 ± 0,068	1,146 ± 0,086	**1,736 ± 0,270**
Aconitase 2	85 kDa	1,000 ± 0,070	1,087 ± 0,015	0,943 ± 0,103	0,997 ± 0,057	1,229 ± 0,137	0,966 ± 0,027	0,755 ± 0,085	1,363 ± 0,305	**1,173 ± 0,109**	0,717 ± 0,103	**1,469 ± 0,287**	**1,641 ± 0,281**
Isocitrate Dehydrogenase 1	46 kDa	1,000 ± 0,207	1,060 ± 0,170	1,137 ± 0,122	1,466 ± 0,214	1,086 ± 0,090	1,289 ± 0,209	1,309 ± 0,210	0,917 ± 0,122	1,111 ± 0,162	1,275 ± 0,297	0,799 ± 0,160	0,931 ± 0,020
Isocitrate Dehydrogenase 2	43 kDa	1,000 ± 0,101	**1,302 ± 0,049**	0,934 ± 0,119	0,785 ± 0,172	1,524 ± 0,438	0,838 ± 0,402	0,624 ± 0,279	**1,792 ± 0,142**	0,684 ± 0,292	1,115 ± 0,152	**2,872 ± 0,527**	**2,008 ± 0,157**
DLST	50 kDa	1,000 ± 0,145	1,295 ± 0,055	0,710 ± 0,093	0,880 ± 0,087	1,016 ± 0,285	**0,375 ± 0,164**	0,324 ± 0,112	**1,361 ± 0,070**	0,546 ± 0,155	0,347 ± 0,157	**3,195 ± 0,569**	2,326 ± 0,752
Fumarase	49 kDa	1,000 ± 0,151	1,064 ± 0,130	0,765 ± 0,053	0,565 ± 0,100	**1,274 ± 0,291**	0,564 ± 0,190	0,245 ± 0,039	**1,396 ± 0,421**	0,497 ± 0,165	0,193 ± 0,047	**1,972 ± 0,623**	**1,707 ± 0,687**
Citrate Synthase	45 kDa	1,000 ± 0,104	1,053 ± 0,037	0,995 ± 0,166	0,840 ± 0,095	1,113 ± 0,120	0,660 ± 0,059	0,455 ± 0,028	**0,961 ± 0,124**	0,594 ± 0,093	0,282 ± 0,069	**1,095 ± 0,254**	**0,911 ± 0,223**
Mitochondrial Pyruvate Carrier 1	12 kDa	1,000 ± 0,186	1,224 ± 0,157	1,086 ± 0,159	1,180 ± 0,007	1,144 ± 0,078	**0,973 ± 0,091**	0,861 ± 0,113	1,253 ± 0,254	1,126 ± 0,082	1,118 ± 0,090	1,569 ± 0,605	1,828 ± 0,362
Mitochondrial Pyruvate Carrier 2	14 kDa	1,000 ± 0,341	0,877 ± 0,161	0,517 ± 0,098	1,187 ± 0,389	0,803 ± 0,121	0,704 ± 0,268	0,767 ± 0,236	0,621 ± 0,103	0,570 ± 0,319	0,934 ± 0,403	0,638 ± 0,181	1,733 ± 0,256
Cytochrome c	14 kDa	1,000 ± 0,267	1,290 ± 0,417	0,939 ± 0,143	0,743 ± 0,301	1,196 ± 0,239	0,680 ± 0,422	0,593 ± 0,384	1,716 ± 0,638	0,598 ± 0,255	0,588 ± 0,234	**3,884 ± 1,297**	**1,732 ± 0,130**
Prohibitin 1	32 kDa	1,000 ± 0,281	1,029 ± 0,174	0,782 ± 0,040	1,102 ± 0,198	**0,456 ± 0,121**	**0,384 ± 0,103**	0,548 ± 0,055	**0,200 ± 0,095**	0,506 ± 0,135	0,469 ± 0,079	0,266 ± 0,131	**0,997 ± 0,164**
COX IV	17 kDa	1,000 ± 0,032	0,865 ± 0,075	**0,855 ± 0,050**	0,712 ± 0,036	**0,442 ± 0,063**	**0,544 ± 0,048**	0,562 ± 0,051	**0,972 ± 0,056**	**1,499 ± 0,245**	0,444 ± 0,016	**1,038 ± 0,027**	**1,280 ± 0,111**
Pyruvate Dehydrogenase	43 kDa	1,000 ± 0,052	0,982 ± 0,143	0,916 ± 0,011	0,561 ± 0,150	**1,009 ± 0,126**	0,375 ± 0,140	0,416 ± 0,215	0,541 ± 0,244	0,291 ± 0,145	0,559 ± 0,142	0,901 ± 0,125	0,593 ± 0,324
SDHA	70 kDa	1,000 ± 0,030	**1,225 ± 0,095**	0,803 ± 0,350	0,931 ± 0,111	**1,389 ± 0,121**	0,794 ± 0,133	0,465 ± 0,154	**1,305 ± 0,181**	0,812 ± 0,196	0,432 ± 0,117	**1,487 ± 0,449**	**1,682 ± 0,240**
HSP60	60 kDa	1,000 ± 0,318	1,134 ± 0,264	0,711 ± 0,228	0,616 ± 0,165	0,832 ± 0,235	**0,204 ± 0,053**	0,222 ± 0,014	0,347 ± 0,378	0,175 ± 0,057	0,131 ± 0,030	0,194 ± 0,099	0,067 ± 0,025
VDAC	32 kDa	1,000 ± 0,191	0,902 ± 0,266	0,699 ± 0,249	0,921 ± 0,214	**0,193 ± 0,164**	0,310 ± 0,234	0,391 ± 0,216	0,144 ± 0,119	0,624 ± 0,289	0,718 ± 0,219	0,248 ± 0,198	1,822 ± 0,709
Acetyl-CoA Carboxylase	280 kDa	1,000 ± 0,164	1,194 ± 0,113	0,967 ± 0,138	1,117 ± 0,123	1,691 ± 0,844	1,062 ± 0,312	1,642 ± 0,275	**0,660 ± 0,107**	0,899 ± 0,664	0,871 ± 0,192	**0,141 ± 0,113**	**0,080 ± 0,079**
P-Acetyl-CoA Carboxylase	280 kDa	1,000 ± 0,066	3,982 ± 0,828	2,743 ± 0,578	2,061 ± 0,175	6,897 ± 0,730	1,774 ± 0,194	1,222 ± 0,084	3,435 ± 0,512	0,698 ± 0,139	0,728 ± 0,072	0,328 ± 0,066	**0,049 ± 0,010**
ATP-Citrate Lyase	125 kDa	1,000 ± 0,021	1,113 ± 0,166	1,035 ± 0,058	0,766 ± 0,288	1,156 ± 0,228	0,791 ± 0,283	0,839 ± 0,178	1,065 ± 0,097	1,010 ± 0,046	0,741 ± 0,301	0,887 ± 0,161	0,819 ± 0,211
P-ATP-Citrate Lyase	125 kDa	1,000 ± 0,318	**2,126 ± 0,450**	0,944 ± 0,230	0,848 ± 0,509	**2,521 ± 0,559**	0,577 ± 0,284	1,156 ± 0,578	3,022 ± 1,339	0,704 ± 0,384	1,175 ± 0,545	1,706 ± 1,001	0,251 ± 0,140
Acetyl-CoA-Synthetase	78 kDa	1,000 ± 0,105	1,169 ± 0,114	1,267 ± 0,199	1,150 ± 0,160	**1,994 ± 0,210**	**1,783 ± 0,202**	1,103 ± 0,189	**1,869 ± 0,184**	1,720 ± 0,341	1,216 ± 0,197	1,697 ± 0,166	**1,907 ± 0,184**
Acyl-CoA-Synthetase	78 kDa	1,000 ± 0,223	1,048 ± 0,133	0,989 ± 0,213	1,002 ± 0,208	1,000 ± 0,196	0,957 ± 0,252	1,003 ± 0,207	0,958 ± 0,246	1,098 ± 0,054	1,097 ± 0,070	1,314 ± 0,230	1,427 ± 0,390
Fatty Acid Synthase	273 kDa	1,000 ± 0,239	1,072 ± 0,164	1,137 ± 0,077	0,990 ± 0,306	1,244 ± 0,070	1,216 ± 0,035	0,952 ± 0,330	1,098 ± 0,141	1,128 ± 0,064	0,903 ± 0,387	1,001 ± 0,267	1,083 ± 0,161
ELOVL6	28 kDa	1,000 ± 0,100	0,964 ± 0,110	0,908 ± 0,184	1,465 ± 0,126	**1,017 ± 0,039**	**0,777 ± 0,175**	1,633 ± 0,189	**0,833 ± 0,104**	**0,439 ± 0,138**	2,592 ± 0,454	**1,000 ± 0,190**	**0,561 ± 0,148**
