# Supplementary material for: Energy Metabolism and Lipidome Are Highly Regulated during Osteogenic Differentiation of Dental Follicle Cells
Source: Stem Cells Int. 2022 Jul 16;2022:3674931. doi: 10.1155/2022/3674931 (PMC9315453; doi:10.1155/2022/3674931)
Supplement: Supplementary 3 — Regulation of less abundant lipid classes during osteogenic differentiation of DFCs. DFCs were cultured in osteogenic differentiation medium (ODM), BMP2 differentiation medium, or control medium (DMEM) for one, seven, 14, and 28 days before the lipidome was analyzed by MS. This figure shows the fraction of lysophosphatidylethanolamines (LPE, a), lysophosphatidylcholines (LPC, b), ceramides (Cer, c), hexosylceramides (HexCer, d), and phosphatidylglycerols (PG, e). Data points show means ± standard deviation, and Student's t-test was performed to compare differentiation medium with control medium at the same time point. ∗p < 0.05, ∗∗p < 0.01, ∗∗∗p < 0.001. Asterisks relate to the differentiation medium with data points and line in same color. [file 3674931.f3.pdf]

Supplementary Figure S1

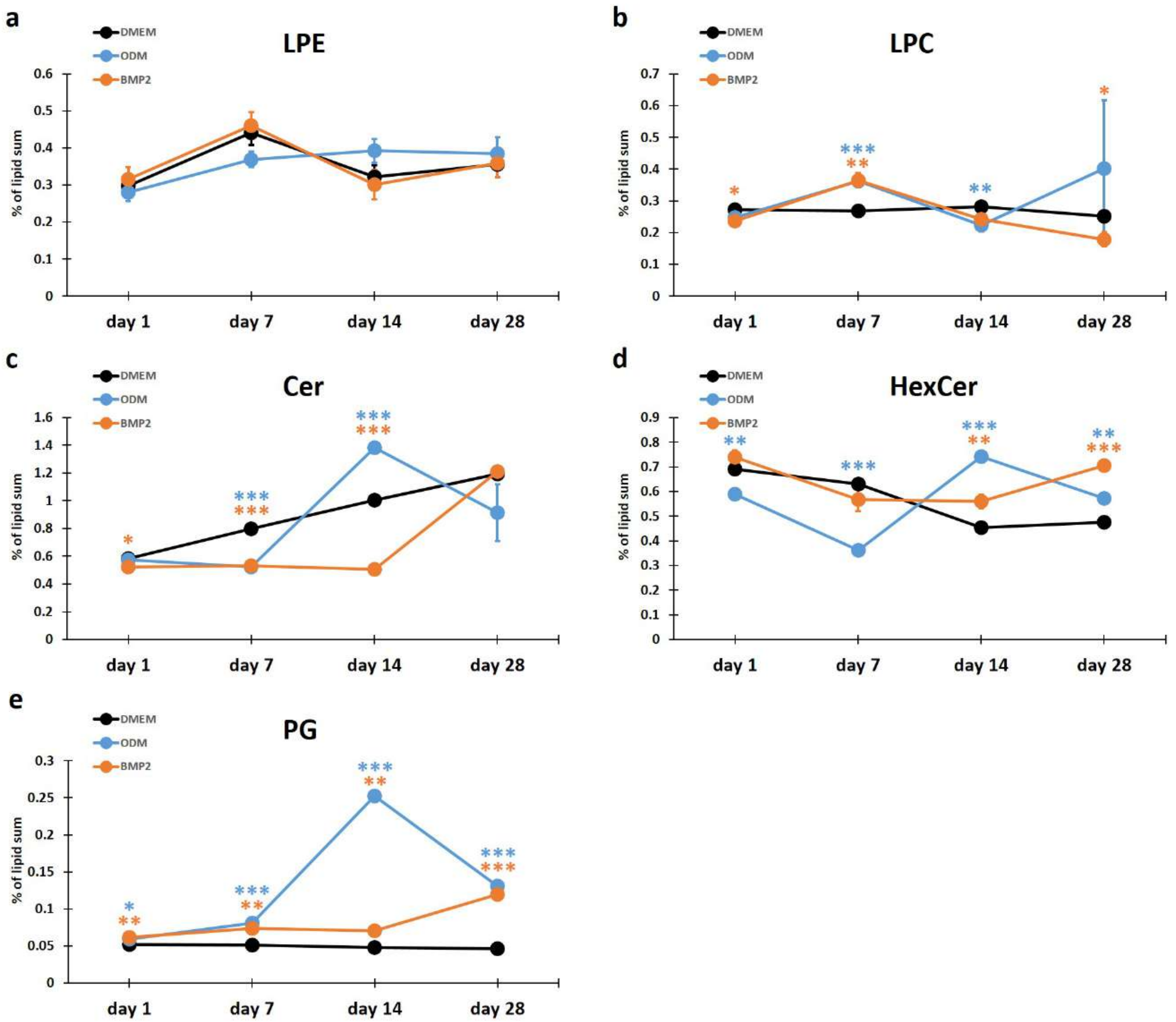

Supplementary Figure S2

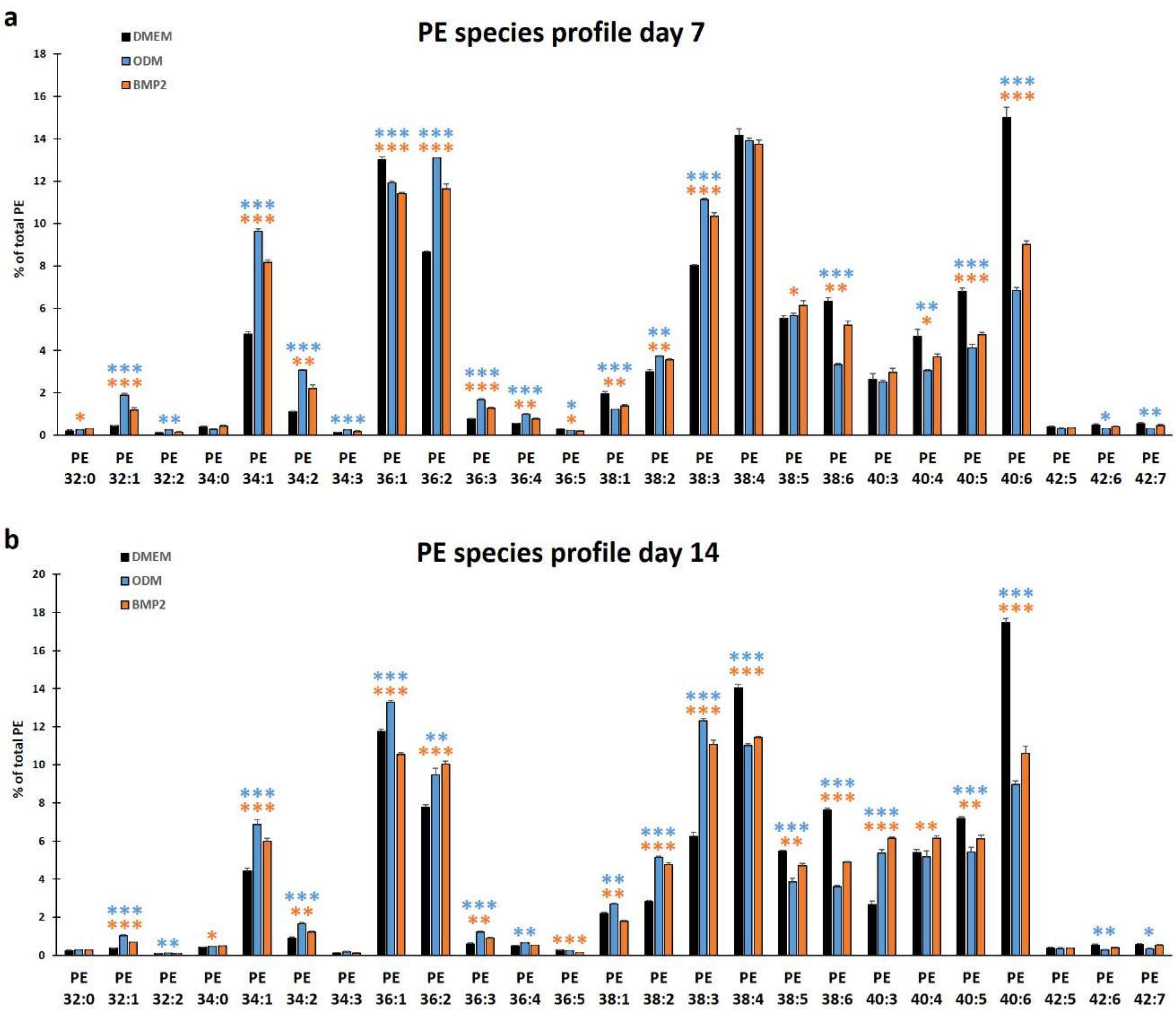

Supplementary Figure S3

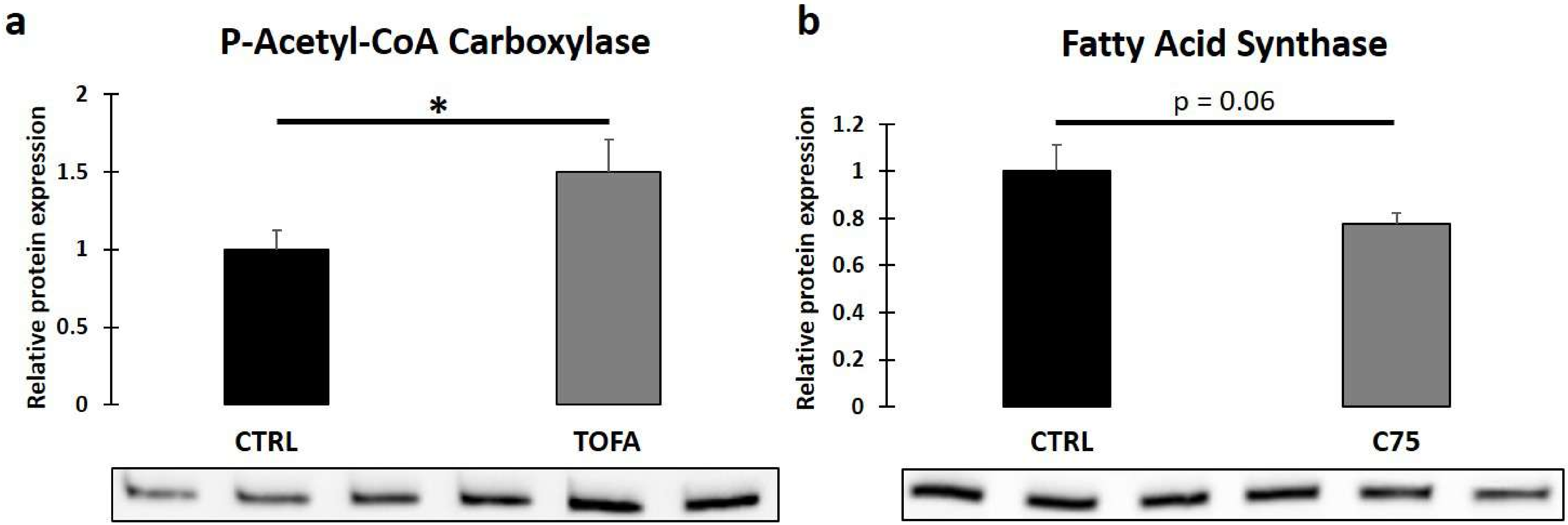

Supplementary Table 1

Upregulated	Downregulated
AKAP5, ASPM, ASPN, CCDC102B, CCNA2, CCNB1, CDKN3, CENPF, CHL1, CNIH3, COL3A1, COLEC10, CORIN, CRISPLD2, CTSK, CYSLTR1, DIO2, DLG7, DPT, EGR1, ENTPD1, ETV1, FOXM1, FRAS1, FRZB, GPRC5B, GPSM2, IFI44L, IFITM1, IL7, KIF20A, MMP1, MOXD1, OAS2, OLFML2B, PLXNC1, PRC1, PTN, RAB27B, RAB31, RGS4, SEPP1, SLC38A4, SLC7A8, SPON2, SPRY1, TMEM60, TRPA1	AARS, ADAMTS5, ALDH1A3, ALDH2, APOL6, ARG2, ARHGEF2, ASNS, B3GALT2, CARS, CBS, CCND2, CDH13, CDRT1, CEBPG, CHAC1, CLDN1, CLSTN2, COL15A1, CTH, CYFIP2, DDIT3, DHRS3, EID3, EIF4EBP1, ELOVL2, FAM129A, FBXW10, FLJ21075, GALNT12, **GOT1**, **GPT2**, GRPR, GUCY1A2, IGFBP5, KCNE4, KCNT2, KIAA0746, KRTAP1-5, LIF, MARS, MSC, MSTN, MTHFD2, NCAM1, OCLN, ODZ2, PAPPA, PAPPA2, PCDH19, PCK2, PDE1C, **PHGDH**, **PSAT1**, **PSPH**, QSOX1, RASSF2, SAV1, SEMA3B, SESN2, **SHMT2**, SLC1A4, SLC1A5, SLC22A15, SLC2A1, SLC2A12, SLC3A2, SLC6A9, SLC7A1, SLC7A11, SLC7A5, SNORD63, SRXN1, STAC, STC2, TCEA1, TM4SF20, TNFSF15, TNFSF18, TNFSF4, TPD52L1, TRIB3, TRIM16L, TUBE1, TUFT1, UHRF1BP1, VEGFA, VLDLR, WARS, YARS

Supplementary Table 2

Antigen	Molecular weight	DMEM 1d	ODM 1d	BMP2 1d	DMEM 7d	ODM 7d	BMP2 7d	DMEM 14d	ODM 14d	BMP2 14d	DMEM 28d	ODM 28d	BMP2 28d
Hexokinase I	102 kDa	1,000 ± 0,294	1,151 ± 0,249	0,901 ± 0,199	0,964 ± 0,104	1,402 ± 0,223	0,831 ± 0,185	0,584 ± 0,201	**1,203 ± 0,123**	0,543 ± 0,142	0,383 ± 0,115	**1,698 ± 0,285**	**1,469 ± 0,422**
Hexokinase II	102 kDa	1,000 ± 0,185	0,731 ± 0,106	0,869 ± 0,205	0,185 ± 0,064	0,251 ± 0,051	0,089 ± 0,039	0,240 ± 0,086	0,226 ± 0,094	0,213 ± 0,046	0,151 ± 0,054	0,300 ± 0,072	0,372 ± 0,248
Phosphofructo-kinase	80 kDa	1,000 ± 0,143	1,090 ± 0,091	0,943 ± 0,058	0,460 ± 0,072	0,611 ± 0,067	0,401 ± 0,036	0,320 ± 0,051	**0,512 ± 0,042**	0,304 ± 0,051	0,201 ± 0,034	**0,546 ± 0,084**	0,342 ± 0,075
GAPDH	37 kDa	1,000 ± 0,201	0,929 ± 0,057	1,050 ± 0,086	1,084 ± 0,058	0,892 ± 0,131	1,215 ± 0,294	0,999 ± 0,251	0,688 ± 0,123	1,074 ± 0,184	0,993 ± 0,254	0,787 ± 0,110	1,292 ± 0,299
Pyruvate Kinase M1/2	60 kDa	1,000 ± 0,268	1,030 ± 0,220	1,179 ± 0,274	1,436 ± 0,368	1,133 ± 0,174	1,693 ± 0,374	1,312 ± 0,300	0,882 ± 0,073	1,332 ± 0,202	1,198 ± 0,245	0,938 ± 0,087	1,168 ± 0,103
Pyruvate Kinase M2	60 kDa	1,000 ± 0,128	1,001 ± 0,084	0,981 ± 0,220	1,070 ± 0,179	0,993 ± 0,152	1,084 ± 0,274	0,912 ± 0,217	0,809 ± 0,023	0,950 ± 0,318	0,763 ± 0,303	0,986 ± 0,160	1,527 ± 0,294
Lactate Dehydrogenase A	37 kDa	1,000 ± 0,122	1,111 ± 0,138	1,119 ± 0,144	1,154 ± 0,089	1,235 ± 0,026	1,383 ± 0,124	1,119 ± 0,055	1,183 ± 0,084	**1,413 ± 0,102**	1,060 ± 0,068	1,146 ± 0,086	**1,736 ± 0,270**
Aconitase 2	85 kDa	1,000 ± 0,070	1,087 ± 0,015	0,943 ± 0,103	0,997 ± 0,057	1,229 ± 0,137	0,966 ± 0,027	0,755 ± 0,085	1,363 ± 0,305	**1,173 ± 0,109**	0,717 ± 0,103	**1,469 ± 0,287**	**1,641 ± 0,281**
Isocitrate Dehydrogenase 1	46 kDa	1,000 ± 0,207	1,060 ± 0,170	1,137 ± 0,122	1,466 ± 0,214	1,086 ± 0,090	1,289 ± 0,209	1,309 ± 0,210	0,917 ± 0,122	1,111 ± 0,162	1,275 ± 0,297	0,799 ± 0,160	0,931 ± 0,020
Isocitrate Dehydrogenase 2	43 kDa	1,000 ± 0,101	**1,302 ± 0,049**	0,934 ± 0,119	0,785 ± 0,172	1,524 ± 0,438	0,838 ± 0,402	0,624 ± 0,279	**1,792 ± 0,142**	0,684 ± 0,292	1,115 ± 0,152	**2,872 ± 0,527**	**2,008 ± 0,157**
DLST	50 kDa	1,000 ± 0,145	1,295 ± 0,055	0,710 ± 0,093	0,880 ± 0,087	1,016 ± 0,285	**0,375 ± 0,164**	0,324 ± 0,112	**1,361 ± 0,070**	0,546 ± 0,155	0,347 ± 0,157	**3,195 ± 0,569**	**2,326 ± 0,752**
Fumarase	49 kDa	1,000 ± 0,151	1,064 ± 0,130	0,765 ± 0,053	0,565 ± 0,100	**1,274 ± 0,291**	0,564 ± 0,190	0,245 ± 0,039	**1,396 ± 0,421**	0,497 ± 0,165	0,193 ± 0,047	**1,972 ± 0,623**	**1,707 ± 0,687**
Citrate Synthase	45 kDa	1,000 ± 0,104	1,053 ± 0,037	0,995 ± 0,166	0,840 ± 0,095	1,113 ± 0,120	0,660 ± 0,059	0,455 ± 0,028	**0,961 ± 0,124**	0,594 ± 0,093	0,282 ± 0,069	**1,095 ± 0,254**	**0,911 ± 0,223**
Mitochondrial Pyruvate Carrier 1	12 kDa	1,000 ± 0,186	1,224 ± 0,157	1,086 ± 0,159	1,180 ± 0,007	1,144 ± 0,078	**0,973 ± 0,091**	0,861 ± 0,113	1,253 ± 0,254	1,126 ± 0,082	1,118 ± 0,090	1,569 ± 0,605	1,828 ± 0,362
Mitochondrial Pyruvate Carrier 2	14 kDa	1,000 ± 0,341	0,877 ± 0,161	0,517 ± 0,098	1,187 ± 0,389	0,803 ± 0,121	0,704 ± 0,268	0,767 ± 0,236	0,621 ± 0,103	0,570 ± 0,319	0,934 ± 0,403	0,638 ± 0,181	1,733 ± 0,256
Cytochrome c	14 kDa	1,000 ± 0,267	1,290 ± 0,417	0,939 ± 0,143	0,743 ± 0,301	1,196 ± 0,239	0,680 ± 0,422	0,593 ± 0,384	1,716 ± 0,638	0,598 ± 0,255	0,588 ± 0,234	**3,884 ± 1,297**	**1,732 ± 0,130**
Prohibitin 1	32 kDa	1,000 ± 0,281	1,029 ± 0,174	0,782 ± 0,040	1,102 ± 0,198	**0,456 ± 0,121**	**0,384 ± 0,103**	0,548 ± 0,055	**0,200 ± 0,095**	0,506 ± 0,135	0,469 ± 0,079	0,266 ± 0,131	**0,997 ± 0,164**
COX IV	17 kDa	1,000 ± 0,032	0,865 ± 0,075	**0,855 ± 0,050**	0,712 ± 0,036	**0,442 ± 0,063**	**0,544 ± 0,048**	0,562 ± 0,051	**0,972 ± 0,056**	**1,499 ± 0,245**	0,444 ± 0,016	**1,038 ± 0,027**	**1,280 ± 0,111**
Pyruvate Dehydrogenase	43 kDa	1,000 ± 0,052	0,982 ± 0,143	0,916 ± 0,011	0,561 ± 0,150	**1,009 ± 0,126**	0,375 ± 0,140	0,416 ± 0,215	0,541 ± 0,244	0,291 ± 0,145	0,559 ± 0,142	0,901 ± 0,125	0,593 ± 0,324
SDHA	70 kDa	1,000 ± 0,030	**1,225 ± 0,095**	0,803 ± 0,350	0,931 ± 0,111	**1,389 ± 0,121**	0,794 ± 0,133	0,465 ± 0,154	**1,305 ± 0,181**	0,812 ± 0,196	0,432 ± 0,117	**1,487 ± 0,449**	**1,682 ± 0,240**
HSP60	60 kDa	1,000 ± 0,318	1,134 ± 0,264	0,711 ± 0,228	0,616 ± 0,165	0,832 ± 0,235	**0,204 ± 0,053**	0,222 ± 0,014	0,347 ± 0,378	0,175 ± 0,057	0,131 ± 0,030	0,194 ± 0,099	0,067 ± 0,025
VDAC	32 kDa	1,000 ± 0,191	0,902 ± 0,266	0,699 ± 0,249	0,921 ± 0,214	**0,193 ± 0,164**	0,310 ± 0,234	0,391 ± 0,216	0,144 ± 0,119	0,624 ± 0,289	0,718 ± 0,219	0,248 ± 0,198	1,822 ± 0,709
Acetyl-CoA Carboxylase	280 kDa	1,000 ± 0,164	1,194 ± 0,113	0,967 ± 0,138	1,117 ± 0,123	1,691 ± 0,844	1,062 ± 0,312	1,642 ± 0,275	**0,660 ± 0,107**	0,899 ± 0,664	0,871 ± 0,192	**0,141 ± 0,113**	**0,080 ± 0,079**
P-Acetyl-CoA Carboxylase	280 kDa	1,000 ± 0,066	3,982 ± 0,828	2,743 ± 0,578	2,061 ± 0,175	6,897 ± 0,730	1,774 ± 0,194	1,222 ± 0,084	3,435 ± 0,512	0,698 ± 0,139	0,728 ± 0,072	0,328 ± 0,066	**0,049 ± 0,010**
ATP-Citrate Lyase	125 kDa	1,000 ± 0,021	1,113 ± 0,166	1,035 ± 0,058	0,766 ± 0,288	1,156 ± 0,228	0,791 ± 0,283	0,839 ± 0,178	1,065 ± 0,097	1,010 ± 0,046	0,741 ± 0,301	0,887 ± 0,161	0,819 ± 0,211
P-ATP-Citrate Lyase	125 kDa	1,000 ± 0,318	**2,126 ± 0,450**	0,944 ± 0,230	0,848 ± 0,509	**2,521 ± 0,559**	0,577 ± 0,284	1,156 ± 0,578	3,022 ± 1,339	0,704 ± 0,384	1,175 ± 0,545	1,706 ± 1,001	0,251 ± 0,140
Acetyl-CoA-Synthetase	78 kDa	1,000 ± 0,105	1,169 ± 0,114	1,267 ± 0,199	1,150 ± 0,160	**1,994 ± 0,210**	**1,783 ± 0,202**	1,103 ± 0,189	**1,869 ± 0,184**	1,720 ± 0,341	1,216 ± 0,197	1,697 ± 0,166	**1,907 ± 0,184**
Acyl-CoA-Synthetase	78 kDa	1,000 ± 0,223	1,048 ± 0,133	0,989 ± 0,213	1,002 ± 0,208	1,000 ± 0,196	0,957 ± 0,252	1,003 ± 0,207	0,958 ± 0,246	1,098 ± 0,054	1,097 ± 0,070	1,314 ± 0,230	1,427 ± 0,390
Fatty Acid Synthase	273 kDa	1,000 ± 0,239	1,072 ± 0,164	1,137 ± 0,077	0,990 ± 0,306	1,244 ± 0,070	1,216 ± 0,035	0,952 ± 0,330	1,098 ± 0,141	1,128 ± 0,064	0,903 ± 0,387	1,001 ± 0,267	1,083 ± 0,161
ELOVL6	28 kDa	1,000 ± 0,100	0,964 ± 0,110	0,908 ± 0,184	1,465 ± 0,126	**1,017 ± 0,039**	**0,777 ± 0,175**	1,633 ± 0,189	**0,833 ± 0,104**	**0,439 ± 0,138**	2,592 ± 0,454	**1,000 ± 0,190**	**0,561 ± 0,148**
