# Supplementary figures and images for: Energy Metabolism and Lipidome Are Highly Regulated during Osteogenic Differentiation of Dental Follicle Cells
Source: Stem Cells Int. 2022 Jul 16;2022:3674931. doi: 10.1155/2022/3674931 (PMC9315453; doi:10.1155/2022/3674931)

# Supplementary Figure S2

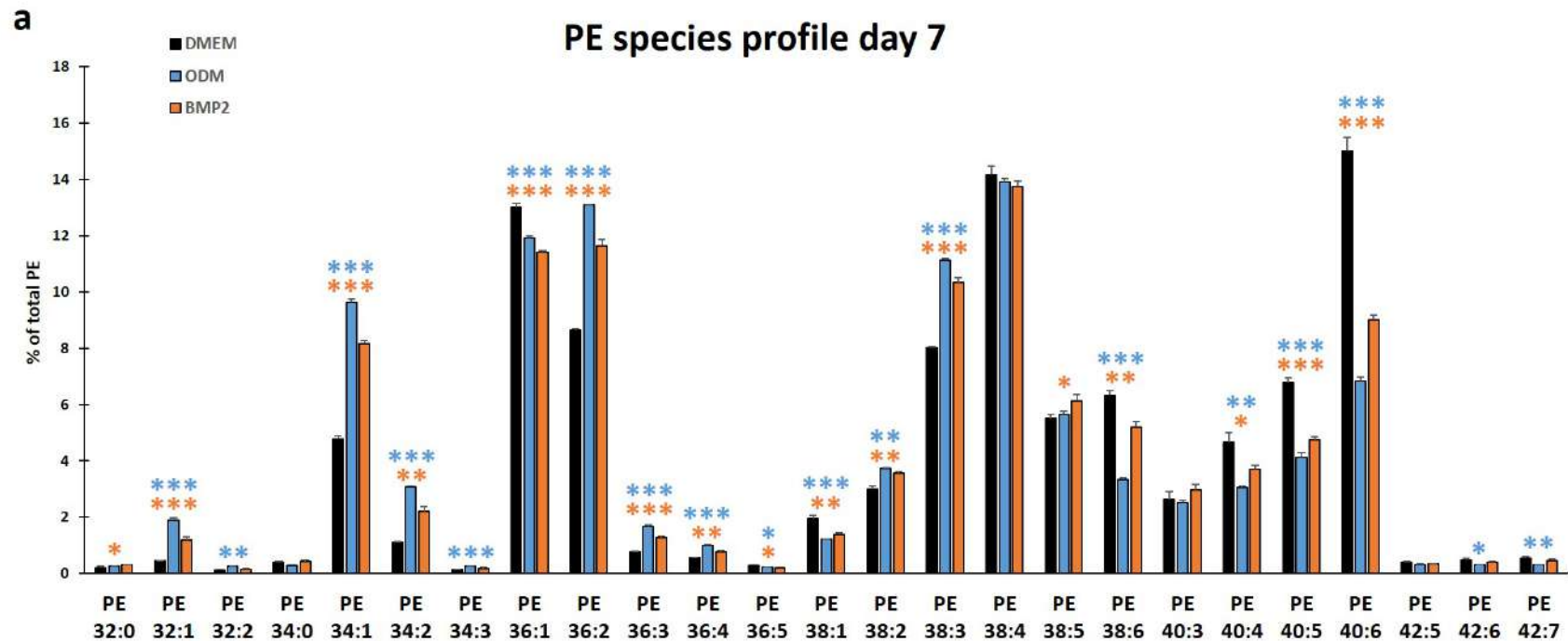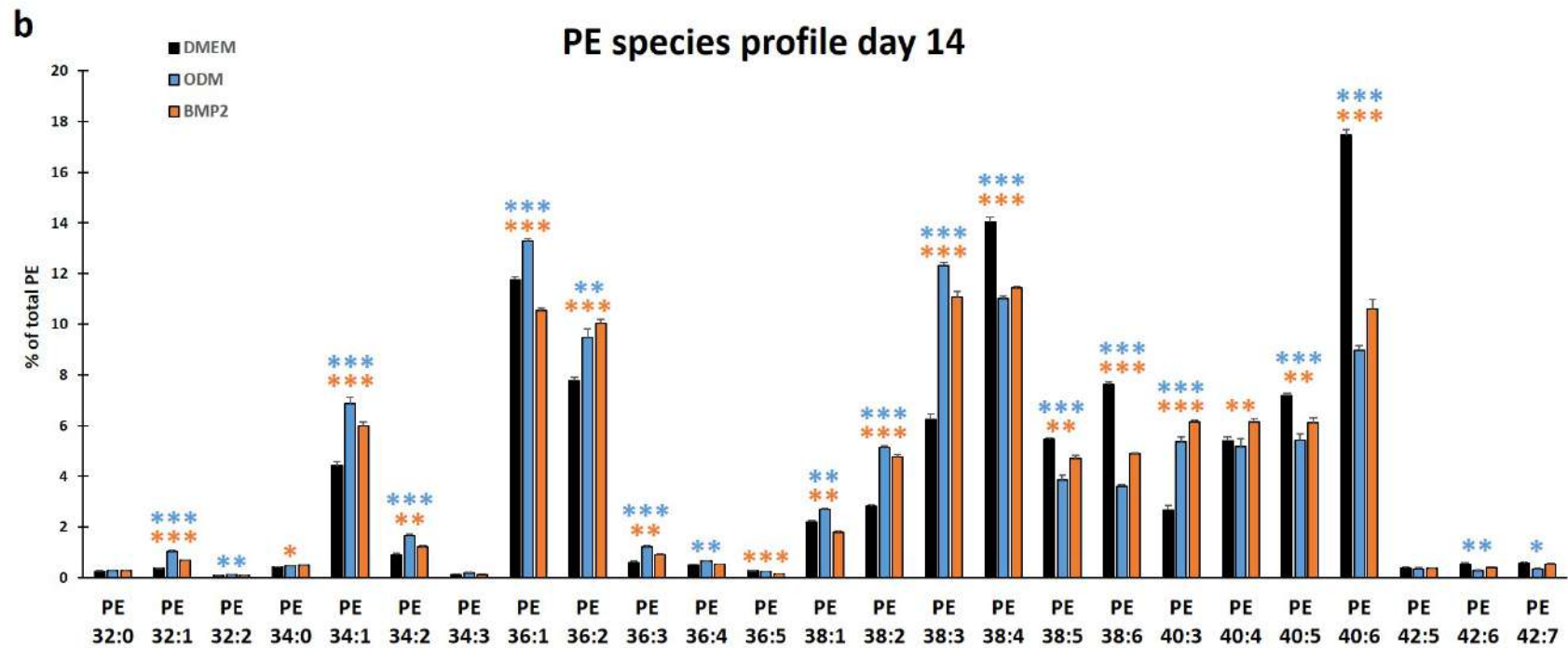

Supplement: Supplementary 4 — Species profiles of phosphatidylethanolamines during osteogenic differentiation of DFCs. This figure shows the composition of phosphatidylethanolamine (PE) species analyzed by MS in DFCs cultured in osteogenic differentiation medium (ODM), BMP2 differentiation medium, or control medium (DMEM) at days 7 (a) and 14 (b). Results are shown as means + standard deviation, and Student's t-test was performed to compare differentiation medium with control medium at the same time point. ∗p < 0.05, ∗∗p < 0.01, ∗∗∗p < 0.001. Asterisks relate to the differentiation medium with bars in same color. [file 3674931.f4.pdf]

# Supplementary Figure S3

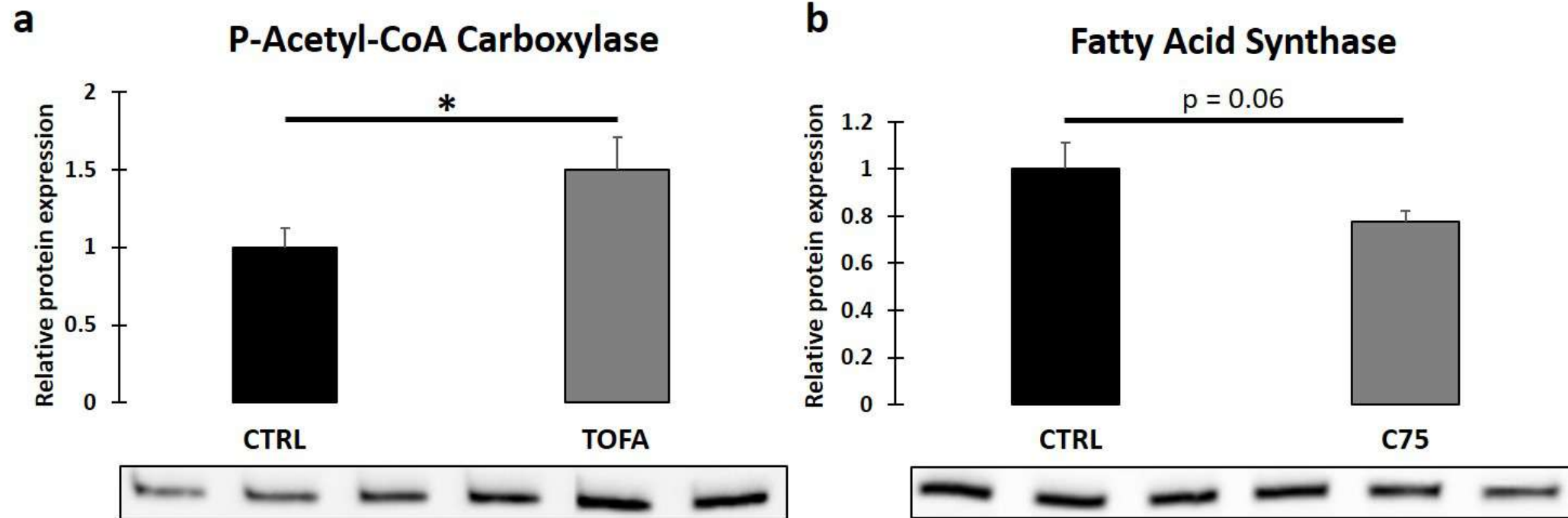

Supplement: Supplementary 5 — Inhibition of fatty acid synthesis by TOFA and C75 in DFCs. DFCs were treated with 5 μg/ml fatty acid synthesis inhibitors TOFA (a) or C75 (b) or vehicle control for two hours (a) or one day (b) before protein expression of phospho-acetyl-CoA carboxylase (a) or fatty acid synthase (b) was determined by western blot analysis. Results are shown as means + standard deviation, and Student's t-test was performed to compare inhibitor treatment with the vehicle control. ∗p < 0.05. [file 3674931.f5.pdf]
